# ﻿Molecular phylogeny of *Nipponacmea* (Patellogastropoda, Lottiidae) from Japan: a re-evaluation of species taxonomy and morphological diagnosis

**DOI:** 10.3897/zookeys.1087.78193

**Published:** 2022-02-25

**Authors:** Shinnosuke Teruya, Davin H. E. Setiamarga, Tomoyuki Nakano, Takenori Sasaki

**Affiliations:** 1 Okinawa Prefectural Deep Sea Water Research Center, 500-1 Maja, Kumejima-cho, Okinawa 901-3104, Japan Okinawa Prefectural Deep Sea Water Research Center Okinawa Japan; 2 The University Museum, The University of Tokyo, 7-3-1 Hongo, Bunkyo-ku, Tokyo 113-0033, Japan The University of Tokyo Tokyo Japan; 3 Department of Applied Chemistry and Biochemistry / Ecosystem Engineering, National Institute of Technology (KOSEN), Wakayama College, 77 Noshima, Nada-cho, Gobo-shi, Wakayama, 644-0023, Japan Department of Applied Chemistry and Biochemistry / Ecosystem Engineering, National Institute of Technology (KOSEN) Wakayama Japan; 4 Seto Marine Biological Laboratory, Field Science Education and Research Centre, Kyoto University, 459 Shirahama, Wakayama, 649-2211, Japan Kyoto University Wakayama Japan

**Keywords:** Lottiidae, morphology, *Nipponacmea*, phylogeny, taxonomy

## Abstract

The patellogastropod limpet genus *Nipponacmea* is widely distributed in Japan and adjacent East Asia. Species identification within *Nipponacmea* is challenging due to the high variation in shell morphology. In this study, we examined the taxonomy of this genus represented by nine nominal species from 43 localities (including type localities). Results of the molecular phylogenetic analysis revealed that: (1) *N.gloriosa*, the sole species in this genus inhabiting the subtidal zone, represents the most basal independent branch; (2) the remaining species are divided into two large clades with lower- and higher-apex shell profiles; and (3) the high-apex morphology was derived from the low-apex type. The terminal clades defined using the molecular data were consistent with nine morphospecies and had 100% bootstrap values, strongly supporting the conventional taxonomy of *Nipponacmea*. Although morphological similarities do not always reflect phylogeny, the set of morphological characters used in the current taxonomy were proven to be adequate for diagnosis. In conclusion, this study provided solid evidence to uphold the monophyly of known species of *Nipponacmea* in Japan and demonstrated the usefulness of morphological characters for species diagnosis.

## ﻿Introduction

Limpets belonging to the clade Patellogastropoda are abundant in the intertidal rocky shores globally and are important in marine biology (Branch 1985a, b). Species taxonomy of patellogastropods has historically been based on the morphology of the shell and radula (Pilsbry 1891; Suter 1904; Oliver 1926; Powell 1973; Ponder and Creese 1980). However, identification of the members of this group is difficult due to the simplicity and high variability of shell morphology (Sasaki 1999a, b; Nakano and Spencer 2007; Nakano et al. 2009a). Therefore, corroboration with molecular phylogenetic analysis is required to establish reliable species taxonomy (Koufopanou et al. 1999), and this approach has resulted in the identification of cryptic species or polymorphisms in certain groups (Nakano and Ozawa 2005; Nakano and Spencer 2007; de Aranzamendi et al. 2009; Nakano et al. 2009a; González-Wevar et al. 2011).

Molecular phylogenetic analysis and comparison of morphological characters have previously been performed for limpets with ambiguous taxonomies (*Lottia*: Simison and Lindberg 2003; *Notoacmea*: Nakano and Spencer 2007; Nakano et al. 2009a; *Patella*: Mauro et al. 2003; *Patelloida*: Nakano and Ozawa 2005; *Nacella*: de Aranzamendi et al. 2009; González-Wevar et al. 2011; *Cellana*: Reisser et al. 2011 and 2012). Use of molecular and morphological characters have led to consistent conclusions in most cases in the genera *Lottia*, *Notoacmea*, and *Patelloida*, whereas species monophyly was rejected in *Nacella* and *Cellana* (see above references). The genetic distances within and among species are variable across taxonomic groups. Previous studies have revealed that the genetic distances within species based on the cytochrome oxidase I gene (COI) are estimated to be less than 4%; however, the values are highly variable among species, ranging from 4% to 44.4% (Mauro et al. 2003; Nakano and Ozawa 2005; Nakano and Spencer 2007; Nakano et al. 2009a). Therefore, there is no fixed threshold for species delimitation using genetic distances, and species taxonomy must also be based on the level of continuity of the morphological characters.

COI is used most frequently in molecular phylogenetic analyses at the population and species levels (Mauro et al. 2003; Simison and Lindberg 2003; Nakano and Ozawa 2005; Nakano and Spencer 2007; de Aranzamendi et al. 2009; Nakano et al. 2009a; González-Wevar et al. 2011; Reisser et al. 2011). In addition, phylogenetic estimation has been based on the 12S rRNA (Goldstien et al. 2009), 16S rRNA (Simison and Lindberg 2003; Nakano and Ozawa 2005; Goldstien et al. 2009), cytochrome b mitochondrial gene (Cytb) (de Aranzamendi et al. 2009; Goldstien et al. 2009), and the ITS1 region from nuclear DNA (Nakano and Spencer 2007; Nakano et al. 2009a). Previous studies have shown that COI is a fast-evolving gene that is suitable for investigation of the validity of species designations (Hebert et al. 2003).

Species delineations have been completed by comparing shell morphology (de Aranzamendi et al. 2009) and radulae (Simison and Lindberg 2003; Nakano and Ozawa 2005; Nakano and Spencer 2007; Nakano et al. 2009a), and through quantitative analysis of shell morphometry (Mauro et al. 2003; González-Wevar et al. 2011; Reisser et al. 2012). Determining the morphology of the radula is often considered one of the most effective means for species identification of patellogastropods (Lindberg 1998; Sasaki 1999a; Nakano and Ozawa 2005, 2007); however, the radular character can vary considerably in some species (e.g., *Notoacmeascapha*; Nakano and Spencer 2007). Therefore, species distinction and identification based solely on the radula is not always reliable. Quantitative analysis of shells may not clearly reveal species boundaries since different species of limpets frequently yield similar shapes. Comparative anatomy using features from the entire animal should be used for species recognition in patellogastropods (Lindberg 1988; Sasaki and Okutani 1993, 1994a, b; Sasaki 1999a); however, comprehensive analysis including both anatomical and molecular characteristics has rarely been conducted with this group.

The genus *Nipponacmea* of the family Lottiidae is widely distributed in East Asia (Nakano and Ozawa 2004, 2007; Nakano and Sasaki 2011), and there are nine known species in Japan (Sasaki 2000, 2017), and at least three more species outside of Japan (Christiaens 1980; Chernyshev and Chernova 2002; Chernyshev 2008; Bouchet 2015, see discussion for details). Before the discovery of specific anatomical characteristics and DNA sequences, the taxonomy of the genus was indistinct (Kira 1954; Habe and Kosuge 1967; Kuroda et al. 1971; Okutani and Habe 1975; Nakamura 1986; Asakura and Nishihama 1987; Takada 1992). Problems in taxonomic classification using morphological characteristics were caused by extensive variation of shell morphology within species. Sasaki and Okutani (1993) observed shell morphology and microstructure as well as anatomy in detail and utilized these features to redefine each species of *Nipponacmea*. As a result, new characters were found in the soft parts of the body, such as snout pigmentation, foot and cephalic tentacles, radula, radula sac configuration, and ovary color.

Molecular phylogenetic analyses of *Nipponacmea* have been undertaken by both Nakano and Ozawa (2004, 2007) and Yu et al. (2014). Nakano and Ozawa (2004, 2007) completed a phylogenetic analysis of the entire patellogastropod clade based on the sequences of the COI, 12S rRNA, 16S rRNA, 18S rRNA, and 28S rRNA genes, in which *Nipponacmea* was supported as a monophyletic lineage, independent of *Notoacmea* and *Tectura*. However, the monophyly of each *Nipponacmea* species could not be tested since only a single individual was used of each. Yu et al. (2014) performed identifications by barcoding and phylogeographic analysis of three *Nipponacmea* species in China, using the COI, 28S rRNA, and histone H3 genes. Currently, phylogenetic and taxonomic classification has only been attempted for selected *Nipponacmea* species in Asia.

The purposes of this study were to: (1) assess the taxonomy of *Nipponacmea* species from Japan using an integrative approach, with distance-based and tree-based methods for molecular data, and testing the utility of morphological diagnostic characters using type specimens and sequenced specimens from type localities or adjacent regions; and (2) phylogenetically analyze the relationships among species.

## ﻿Materials and methods

### ﻿Collection of samples

**Figure 1.**
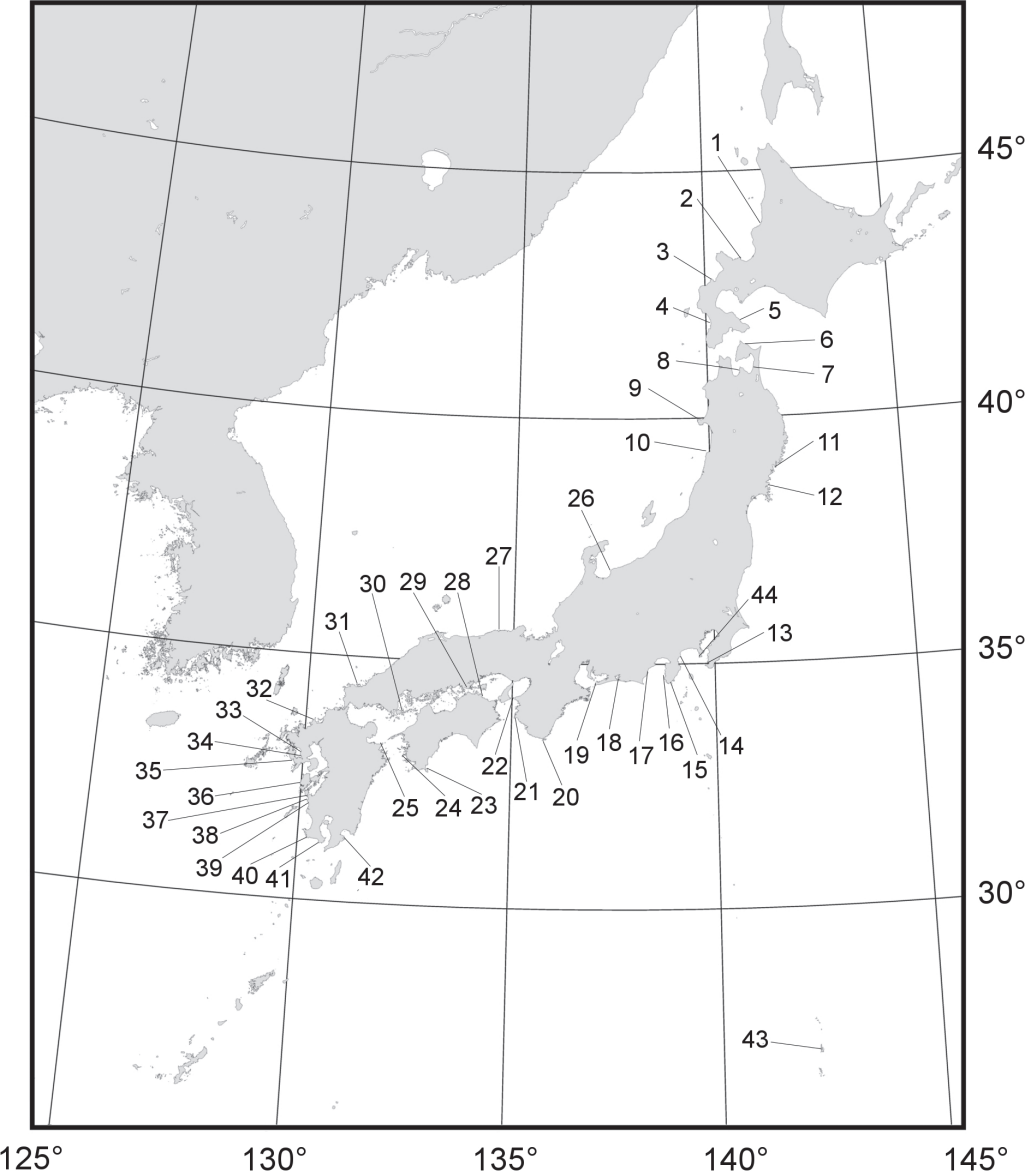
Collection localities of the specimens used in this study. The numbers are shown in Table 1.

We collected *Nipponacmea* samples from 43 localities on the Japanese coast (Fig. 1, Table 1). The type localities or nearby areas are included for nine nominal species in this study (see Table 2). In addition, three species of *Lottia* (*L.kogamogai* (southern population), *L.tenuisculpta*, and *L.lindbergi*) described by Sasaki and Okutani (1994c), were used as outgroups.

**Table 1.** List of localities. See also Fig. 1 for map and Table 2 for list of specimens. All localities are in Japan.

No.	Locality	Coordinates (Latitude, Longitude)
1	Omachi, Rumoi, Hokkaido	43°56'45"N, 141°37'41"E
2	Shukutsu, Otaru, Hokkaido	43°14'09"N, 141°00'57"E
3	Masadomari, Suttu, Hokkaido	42°49'28"N, 140°11'15"E
4	Genna, Otobe, Hokkaido	42°00'24"N, 140°06'15"E
5	Usujiri, Hokkaido	41°56'11"N, 140°56'57"E
6	Hebiura, Kazamaura, Aomori Prefecture	41°29'42"N, 140°58'55"E
7	Arito, Noheji, Aomori Prefecture	40°54'25"N, 141°10'50"E
8	Tsuchiya, Hiranai, Aomori Prefecture	40°54'13"N, 140°51'46"E
9	Togashiohama, Oga, Akita Prefecture	39°56'40"N, 139°42'14"E
10	Kisakata, Nikaho, Akita Prefecture	39°12'34"N, 139°53'34"E
11	Masakicho, Ofunato, Iwate Prefecture	39°01'23"N, 141°42'36"E
12	Karakuwa, Ishinomaki, Miyagi Prefecture	38°30'47"N, 141°28'45"E
13	Okinoshima, Tateyama, Chiba Prefecture	34°59'27"N, 139°49'51"E
14	Mitsuishi, Manazuru, Kanagawa Prefecture	35°08'25"N, 139°09'41"E
15	Irouzaki, Minamiizu, Shizuoka Prefecture	34°36'47"N, 138°50'57"E
16	Futo, Nishiizu, Shizuoka Prefecture	34°47'36"N, 138°45'26"E
17	Iwashigashima, Yaizu, Shizuoka Prefecture	34°51'30"N, 138°19'40"E
18	Yutocho, Hamamatsu, Shizuoka Prefecture	34°42'13"N, 137°36'48"E
19	Iragocho, Tahara, Aichi Prefecture	34°34'56"N, 137°01'01"E
20	Shionomisaki, Kushimoto, Wakayama Prefecutre	33°26'11"N, 135°45'23"E
21	Mio, Mihamacho, Wakayama Prefecture	33°53'15"N, 135°04'31"E
22	Kada, Wakayama Prefecture	34°16'21"N, 135°03'54"E
23	Oki, Tosashimizu, Kochi Prefecture	32°51'00"N, 132°57'21"E
24	Ajiro, Ainancho, Ehime Prefecture	33°02'00"N, 132°24'19"E
25	Ohira, Oita, Oita Prefecture	33°14'50"N, 131°49'40"E
26	Suwacho, Uozu, Toyama Prefecture	36°48'40"N, 137°23'33"E
27	Yoroi, Kazumi, Hyogo Prefecture	35°39'10"N, 134°34'37"E
28	Tsudacho, Sanuki, Kagawa Prefecture	34°17'16"N, 134°16'04"E
29	Shibukawa, Tamano, Okayama Prefecture	34°27'23"N, 133°53'51"E
30	Hirano, Suo-Oshima, Yamaguchi Prefecture	33°53'59"N, 132°21'51"E
31	Higashifukawa, Nagato, Yamaguchi Prefecture	34°22'32"N, 131°10'33"E
32	Nishinoura, Nishi-ku, Fukuoka Prefecture	33°39'20"N, 130°12'28"E
33	Hiranitago, Higashisonogi, Nagasaki Prefecture	33°00'26"N, 129°56'47"E
34	Kujima, Omura, Nagasaki Prefecture	32°53'42"N, 129°57'11"E
35	Nagatamachi, Nagasaki Prefecture	32°50'00"N, 129°43'01"E
36	Odatoko Bay, Amakusa, Kumamoto Prefecture	32°24'07"N, 130°00'09"E
37	Wakimoto, Akune, Kagoshima Prefecture	32°05'03"N, 130°11'26"E
38	Sagata, Akune, Kagoshima Prefecture	31°59'31"N, 130°10'54"E
39	Okawa, Akune, Kagoshima Prefecture	31°56'47"N, 130°12'58"E
40	Bonotsu, Minamisatsuma, Kagoshima Prefecture	31°16'26"N, 130°13'19"E
41	Kaimon, Ibusuki, Kagoshima Prefecture	31°11'28"N, 130°30'30"E
42	Kishira, Kimotsuki, Kagoshima Prefecture	31°13'41"N, 131°01'04"E
43	Chichijima, Ogasawara Islands	27°05'36"N, 142°11'39"E
44	Koajiro, Misaki, Miura, Kanagawa Prefecture	35°09'27"N, 139°36'40"E

Animals were preserved in 99% ethanol. Preliminary identification of specimens prior to DNA sequencing was based on shell characters (Sasaki and Okutani 1993; Sasaki 2000, 2017). All voucher specimens were deposited in the Department of Historical Geology and Paleontology at The University Museum, University of Tokyo (UMUT RM31815–31935, 32353–32364).

**Table 2.** List of specimens used in this study. UMUT: The University Museum, The University of Tokyo. *Type locality, ** locality close to type locality.

Species	UMUT no.	Loc. no. (Fig. 1)	Accession no.				Figure(s)
			COI	Cytb	12S	16S	
*N.boninensis*	RM31815	43*	LC138228	LC142818	LC142951	LC143084	Figs 3N, 7G
	RM31816	43*	LC138229	LC142819	LC142952	LC143085	Figs 3O, 5C
	RM31817	43*	LC138230	LC142820	LC142953	LC143086	Figs 3K–M, 6C, 7F
*N.concinna*	RM31818	10	LC138231	LC142821	LC142954	LC143087	
	RM31819	10	LC138232	LC142822	LC142955	LC143088	
	RM31820	11	LC138233	LC142823	LC142956	LC143089	Fig. 3U–W
	RM31821	11	LC138234	LC142824	LC142957	LC143090	
	RM31822	17	LC138235	LC142825	LC142958	LC143091	
	RM31823	19	LC138236	LC142826	LC142959	LC143092	Fig. 7M
	RM31824	21	LC138237	LC142827	LC142960	LC143093	Fig. 3X
	RM31825	21	LC138238	LC142828	LC142961	LC143094	
	RM31826	29	LC138239	LC142829	LC142962	LC143095	
	RM31827	30	LC138240	LC142830	LC142963	LC143096	
	RM31828	30	LC138241	LC142831	LC142964	LC143097	Fig. 3Y
	RM31829	32	LC138242	LC142832	LC142965	LC143098	
	RM31830	34	LC138243	LC142833	LC142966	LC143099	Fig. 5E
	RM31831	34	LC138244	LC142834	LC142967	LC143100	Fig. 7K
	RM32353	35*	LC138349	LC142939	LC143072	LC143205	Figs 6E, 7L
*N.fuscoviridis*	RM31832	1	LC138245	LC142835	LC142968	LC143101	
	RM31833	1	LC138246	LC142836	LC142969	LC143102	
	RM31834	1	LC138247	LC142837	LC142970	LC143103	Fig. 7E
	RM31835	1	LC138248	LC142838	LC142971	LC143104	
	RM31836	1	LC138249	LC142839	LC142972	LC143105	
	RM31837	4	LC138250	LC142840	LC142973	LC143106	
	RM31838	4	LC138251	LC142841	LC142974	LC143107	
	RM31839	4	LC138252	LC142842	LC142975	LC143108	
	RM31840	8	LC138253	LC142843	LC142976	LC143109	
	RM31841	10	LC138254	LC142844	LC142977	LC143110	
	RM31842	10	LC138255	LC142845	LC142978	LC143111	
	RM31843	10	LC138256	LC142846	LC142979	LC143112	
	RM31844	10	LC138257	LC142847	LC142980	LC143113	
	RM31845	10	LC138258	LC142848	LC142981	LC143114	
	RM31846	10	LC138259	LC142849	LC142982	LC143115	Fig. 3I
	RM31847	13	LC138260	LC142850	LC142983	LC143116	Fig. 5B
	RM31848	32	LC138261	LC142851	LC142984	LC143117	
	RM31849	32	LC138262	LC142852	LC142985	LC143118	
	RM31850	32	LC138263	LC142853	LC142986	LC143119	
	RM31851	32	LC138264	LC142854	LC142987	LC143120	
	RM31852	32	LC138265	LC142855	LC142988	LC143121	
	RM31853	36	LC138266	LC142856	LC142989	LC143122	
	RM31854	36	LC138267	LC142857	LC142990	LC143123	
	RM31855	36	LC138268	LC142858	LC142991	LC143124	
	RM31856	36	LC138269	LC142859	LC142992	LC143125	
	RM31857	39*	LC138270	LC142860	LC142993	LC143126	
	RM32354	39*	LC138350	LC142940	LC143073	LC143206	Figs 6B, 7D
	RM31858	42	LC138271	LC142861	LC142994	LC143127	Figs 3F–H, 7C
	RM31859	42	LC138272	LC142862	LC142995	LC143128	Fig. 3J
*N.gloriosa*	RM31860	13	LC138273	LC142863	LC142996	LC143129	Figs 3D, 7B
	RM31861	14	LC138274	LC142864	LC142997	LC143130	Fig. 5A
	RM31862	14	LC138275	LC142865	LC142998	LC143131	Fig. 3E
	RM31863	14	LC138276	LC142866	LC142999	LC143132	
	RM31864	16	LC138277	LC142867	LC143000	LC143133	
	RM31865	27	LC138278	LC142868	LC143001	LC143134	
	RM31866	27	LC138279	LC142869	LC143002	LC143135	
*N.gloriosa*	RM31867	27	LC138280	LC142870	LC143003	LC143136	
	RM31868	40	LC138281	LC142871	LC143004	LC143137	
	RM31869	41	LC138282	LC142872	LC143005	LC143138	Fig. 3A–C
	RM32355	41	LC138351	LC142941	LC143074	LC143207	Figs 6A, 7A
*N.habei*	RM31870	2	LC138283	LC142873	LC143006	LC143139	Fig. 5H
	RM31871	3	LC138284	LC142874	LC143007	LC143140	
	RM31872	3	LC138285	LC142875	LC143008	LC143141	Fig. 7U
	RM31873	5**	LC138286	LC142876	LC143009	LC143142	Figs 4T, 7V
	RM32357	5**	LC138353	LC142943	LC143076	LC143209	Fig. 7W
	RM31874	12	LC138287	LC142877	LC143010	LC143143	Fig. 4P–R
	RM31875	13	LC138288	LC142878	LC143011	LC143144	Fig. 4S
	RM32356	13	LC138352	LC142942	LC143075	LC143208	Figs 6H, 7X
	RM32364	13	LC138360	LC142950	LC143083	LC143216	Fig. 7T
*N.nigrans*	RM31876	1	LC138289	LC142879	LC143012	LC143145	
	RM31877	3	LC138290	LC142880	LC143013	LC143146	
	RM31878	3	LC138291	LC142881	LC143014	LC143147	
	RM31879	3	LC138292	LC142882	LC143015	LC143148	
	RM31880	3	LC138293	LC142883	LC143016	LC143149	
	RM31881	4	LC138294	LC142884	LC143017	LC143150	
	RM31882	4	LC138295	LC142885	LC143018	LC143151	
	RM31883	7	LC138296	LC142886	LC143019	LC143152	
	RM31884	11	LC138297	LC142887	LC143020	LC143153	
	RM31885	12	LC138298	LC142888	LC143021	LC143154	
	RM31886	15	LC138299	LC142889	LC143022	LC143155	Fig. 4N
	RM31887	15	LC138300	LC142890	LC143023	LC143156	Fig. 4K–M
	RM32358	20*	LC138354	LC142944	LC143077	LC143210	Fig. 7S
	RM32359	20*	LC138355	LC142945	LC143078	LC143211	Fig. 7R
	RM32360	20*	LC138356	LC142946	LC143079	LC143212	Fig. 7Q
	RM32361	20*	LC138357	LC142947	LC143080	LC143213	Fig. 5G
	RM32362	20*	LC138358	LC142948	LC143081	LC143214	Fig. 6G
	RM31888	22	LC138301	LC142891	LC143024	LC143157	
	RM31889	22	LC138302	LC142892	LC143025	LC143158	
	RM31890	22	LC138303	LC142893	LC143026	LC143159	
	RM31891	26	LC138304	LC142894	LC143027	LC143160	
	RM31892	32	LC138305	LC142895	LC143028	LC143161	Fig. 4F–H
	RM31893	33	LC138306	LC142896	LC143029	LC143162	
	RM31894	33	LC138307	LC142897	LC143030	LC143163	
	RM31895	33	LC138308	LC142898	LC143031	LC143164	Fig. 4J
	RM31896	33	LC138309	LC142899	LC143032	LC143165	
	RM31897	33	LC138310	LC142900	LC143033	LC143166	Fig. 4O
*N.radula*	RM31898	18	LC138311	LC142901	LC143034	LC143167	Fig. 7N
	RM31899	31	LC138312	LC142902	LC143035	LC143168	Fig. 4E
	RM31900	31	LC138313	LC142903	LC143036	LC143169	Fig. 5F
	RM31901	34	LC138314	LC142904	LC143037	LC143170	
	RM31902	34	LC138315	LC142905	LC143038	LC143171	Fig. 4D
	RM31903	34	LC138316	LC142906	LC143039	LC143172	
	RM31904	34	LC138317	LC142907	LC143040	LC143173	Figs 4A–C, 7O
	RM32363	37*	LC138359	LC142949	LC143082	LC143215	Figs 6F, 7P
*N.schrenckii*	*RM31905*	6	LC138318	LC142908	LC143041	LC143174	
	RM31906	6	LC138319	LC142909	LC143042	LC143175	Figs 3P–R, 6D, 7I
	RM31907	6	LC138320	LC142910	LC143043	LC143176	
	RM31908	6	LC138321	LC142911	LC143044	LC143177	Figs 3S, 5D
	RM31909	9	LC138322	LC142912	LC143045	LC143178	
	RM31910	9	LC138323	LC142913	LC143046	LC143179	
	RM31911	9	LC138324	LC142914	LC143047	LC143180	
	RM31912	14	LC138325	LC142915	LC143048	LC143181	
*N.schrenckii*	RM31913	14	LC138326	LC142916	LC143049	LC143182	
	RM31914	23	LC138327	LC142917	LC143050	LC143183	
	RM31915	30	LC138328	LC142918	LC143051	LC143184	Fig. 7H
	RM31916	35*	LC138329	LC142919	LC143052	LC143185	Figs 3T, 7J
*N.teramachii*	RM31917	13	LC138330	LC142920	LC143053	LC143186	Fig. 5I
	RM31918	13	LC138331	LC142921	LC143054	LC143187	
	RM31919	21	LC138332	LC142922	LC143055	LC143188	
	RM31920	21	LC138333	LC142923	LC143056	LC143189	
	RM31921	24	LC138334	LC142924	LC143057	LC143190	
	RM31922	24	LC138335	LC142925	LC143058	LC143191	Fig. 4Y
	RM31923	25	LC138336	LC142926	LC143059	LC143192	
	RM31924	25	LC138337	LC142927	LC143060	LC143193	Fig. 7Z
	RM31925	28	LC138338	LC142928	LC143061	LC143194	Fig. 4X
	RM31926	28	LC138339	LC142929	LC143062	LC143195	Fig. 7Y
	RM31927	30	LC138340	LC142930	LC143063	LC143196	
	RM31928	30	LC138341	LC142931	LC143064	LC143197	Fig. 6I
	RM31929	32	LC138342	LC142932	LC143065	LC143198	
	RM31930	32	LC138343	LC142933	LC143066	LC143199	Fig. 4U–W
	RM31931	38*	LC138344	LC142934	LC143067	LC143200	
	RM31932	38*	LC138345	LC142935	LC143068	LC143201	
*L.kogamogai*	RM31933	44	LC138346	LC142936	LC143069	LC143202	
*L.tenuisculpta*	RM31934	44	LC138347	LC142937	LC143070	LC143203	
*L.lindbergi*	RM31935	44	LC138348	LC142938	LC143071	LC143204	

### ﻿DNA extraction, amplification, and sequencing

Total genomic DNA was extracted from the mantle using the cetyltrimethylammonium bromide (CTAB) method (Doyle and Doyle 1990). The mtDNA cytochrome c oxidase I (COI), cytochrome b (Cytb), the small-subunit ribosomal RNA (12S rRNA), and the large-subunit ribosomal RNA (16S rRNA) were used as the molecular markers in this study. PCR products of each gene was amplified with universal primers (Table 3). PCR amplification was performed in a reaction volume of 25 μL containing 10 μM Tris HCl at pH 8.3, 50 μM KCL, 1.5 μM MgCl_2_, 200 μM dNTPs, 0.2 μM of each primer, 2 units of Taq polymerase (Takara), and 1 μL of template DNA. The amplification cycle consisted of an initial denaturation for 3 min at 94 °C, followed by 30 cycles of denaturation for 45 s at 94 °C, annealing for 90 s at a gene-specific annealing temperature (50 °C for COI, 52 °C for Cytb, and 55 °C for the 12S) and extension for 120 s at 72 °C, followed by a 5 min final extension at 72 °C. The PCR products were purified with Illustra ExoStar (GE Healthcare), and used as the template DNA for cycle sequencing reactions from both directions with the DTCS-Quick Start Kit (Beckman Coulter) following standard protocols using the CEQ 2000 XL (Beckman Coulter) automatic sequencer.

**Table 3.** List of PCR primers.

Gene	Primer name	Sequence (5’→3’)	Source
COI	LCO1490 (F)	GGTCAACAAATCATAAAGATATTGG	Folmer et al. (1994)
	HCO2198 (R)	TAAACTTCAGGGTGACCAAAAAATCA	Folmer et al. (1994)
Cytb	cobF (F)	GGWTAYGTWYTWCCWTGRGGWCARAT	Boore and Brown 2000
	cobR (R)	GCRTAWGCRAAWARRAARTAYCAYTCWGG	Boore and Brown 2000
12S	12Sma (F)	CTGGGATTAGATACCCTGTTAT	Koufopanou et al. (1999)
	12Smb (R)	CAGAGAGTGACGGGCGATTTGT	Koufopanou et al. (1999)
16S	16LRN13398 (F)	CGCCTGTTTAACAAAAACAT	Koufopanou et al. (1999)
	16SRHTB (R)	ACGCCGGTTTGAACTCAGATC	Koufopanou et al. (1999)

### ﻿Datasets

All sequences were aligned using MEGA 6.06 (Tamura et al. 2013) and multiple sequence alignments were constructed using MAFFT (Katoh and Toh 2008). Ambiguous regions were removed with Gblocks (Talavera and Castresana 2007) to allow for smaller final blocks and less strict flanking positions.

### ﻿Phylogenetic analyses

Phylogenetic analyses were conducted using a maximum-likelihood (ML) approach via GARLI v. 2.0 (Zwickl 2006) and a Bayesian approach via MrBayes v3.1.2 (Ronquist and Huelsenbeck 2003) with appropriate substitution models for each partition. MrModeltest v2.3 (Nylander 2004) was applied to obtain appropriate substitution models using the Akaike information criterion (Akaike 1974). The substitution models chosen were GTR+I+G for the 12S rRNA, 16S rRNA and Cytb genes, and HKY+I+G for the COI gene.

ML bootstrap values were calculated from 1000 replicates. MrBayes was utilized with the following settings: six substitution types were employed (nst = 6); rate variation across sites was modeled using a gamma distribution with a proportion of the sites as invariant (rate = invgamma); and finally, the shape, invariable site proportion, state frequency, and substitution rate parameters were estimated.

Bayesian analysis was performed for 4,000,000 generations (for the four genes concatenated), 4,500,000 generations (COI), 4,000,000 generations (Cytb), 3,500,000 generations (12S rRNA), and 6,000,000 generations (16S rRNA) with a sample frequency of 100 and the first 25% generations discarded as the burn-in; convergence was determined when the average standard deviation of the split frequencies value (ASDSF) was below 0.01.

The genetic distances among and within species were calculated using the Kimura-2-Parameter (K2P) in MEGA 6.06.

### ﻿Morphological characters

Sequenced specimens were dissected under a binocular microscope. After observations of the animal including the snout pigmentation, cephalic tentacles, and foot lateral wall, the visceral mass was dissected to reveal the configuration of the radular sac. Removed radulae were cleaned in diluted commercial bleach, coated with platinum vanadium, and observed with a scanning electron microscope (Keyence VE-8800). The color of the ovary was recorded before ethanol fixation for specimens collected in breeding season, since gonad color fades when stored in ethanol.

Three shell characters were measured for a total of 130 sequenced specimens: shell length (L), shell width (W), and shell height (H). All individuals were measured with a digital caliper (to 0.01 mm). Allometric analyses were performed among species and genetic groups to determine relationships among length, width, and height using Welch’s t-test. Canonical discriminant analysis was performed among species using the three shell characters (L, W, and H). Discriminant functions also calculated the percentage of individuals that were classified correctly. Canonical discriminant analysis was conducted using R software package version 3.1.0 (R Core Team 2014).

## ﻿Results

### ﻿Molecular data

A total of 130 *Nipponacmea* individuals morphologically identified as *N.schrenckii* (12), *N.fuscoviridis* (29), *N.concinna* (15), *N.radula* (8), *N.boninensis* (3), *N.habei* (9), *N.teramachii* (16), *N.nigrans* (27), and *N.gloriosa* (11) were sequenced (Table 2). The lengths of the COI, Cytb, 12S rRNA, and 16S rRNA gene sequences were 648, 410, 443, and 604 bp, respectively. After removal of ambiguous regions and trimming the ends of poor quality sequences, final lengths of 506, 404, 324, and 575 bp were used for the analysis, respectively. The sequences of the four genes were combined into a total of 1809 bp for constructing phylogenetic trees. All nucleotide sequences in this study were deposited in GenBank (Accession numbers LC138228–LC138360, LC14818–LC143216).

**Table 4.** Genetic distances among *Nipponacmea* species using COI, Cytb, and the 12S rRNA gene. Numbers in bold typeface indicated the intraspecific.

	1	2	3	4	5	6	7	8	9	10
COI										
1 *N.nigrans*	**0.0–5.5**									
2 *N.habei*	21.5–23.7	**0.0–0.8**								
3 *N.teramachii*	22.1–25.1	21.7–22.9	**0.0–0.8**							
4 *N.fuscoviridis*	24.9–28.1	22.1–23.1	22.1–23.1	**0.0–1.2**						
5 *N.boninensis*	23.5–24.7	23.7–24.1	24.1–25.1	19.6–20.8	**0.0–0.4**					
6 *N.schrenckii*	23.1–25.1	22.5–23.1	23.3–24.5	18.6–19.6	17.8–18.4	**0.0–1.0**				
7 *N.concinna*	22.9–24.9	24.3–25.3	23.7–24.3	19.4–20.9	19.6–20.2	20.8–21.9	**0.0–0.8**			
8 *N.radula*	25.1–27.3	23.1–26.9	25.7–26.9	23.3–24.9	18.8–21.7	21.5–23.1	21.7–23.7	**0.0–9.9**		
9 *N.gloriosa*	26.7–29.2	27.5–28.1	26.3–27.5	26.5–27.5	26.9–27.9	26.5–27.7	24.9–26.3	29.4–32	**0.0–0.8**	
10 *L.kogamogai*	25.0–27.0	24.5–24.7	24.7–25.1	25.9–26.9	26.9–27.1	25.7–26.3	25.3–25.9	25.3–27.5	28.1–28.5	**0.0**
Cytb										
1 *N.nigrans*	**0.0–4.7**									
2 *N.habei*	20.5–22.0	**0.0–0.7**								
3 *N.teramachii*	24.8–27.0	23.8–24.5	**0.0–1.2**							
4 *N.fuscoviridis*	23.0–24.8	24.0–24.8	23.3–24.3	**0.0–0.5**						
5 *N.boninensis*	21.3–22.8	20.5–20.8	21.8–22.5	17.1–17.3	**0.0**					
6 *N.schrenckii*	24.8–27.0	22.0–22.8	23.0–24.8	19.8–21.0	21.0–21.8	**0.0–1.0**				
7 *N.concinna*	26.0–27.5	26.2–27.0	23.0–23.8	18.8–19.8	19.1–19.8	21.5–22.3	**0.0–0.7**			
8 *N.radula*	24.5–30.0	22.0–24.0	21.8–22.5	21.0–21.5	21.0–22.3	18.6–20.0	21.8–22.3	**0.0–7.7**		
9 *N.gloriosa*	21.8–23.8	20.3–21.3	23.8–25.2	23.3–24.8	24.0–24.5	23.3–24.3	24.5–26.5	23.5–25.0	**0–2.5**	
10 *L.kogamogai*	26.2–27.0	32.4–32.4	28.2–28.7	28.0–28.2	28.7–28.7	31.2–31.4	29.7–30.2	30.0–30.4	30.2–31.2	**0.0**
12S rRNA										
1 *N.nigrans*	**0.0–1.2**									
2 *N.habei*	10.5–11.1	**0.0**								
3 *N.teramachii*	12.7–13.6	13.0	**0.0**							
4 *N.fuscoviridis*	15.4–16.0	14.8–15.1	14.2–14.5	**0.0–0.3**						
5 *N.boninensis*	16.0–16.7	14.8	14.8	5.6–5.9	**0.0**					
6 *N.schrenckii*	16.0–16.7	14.8	16.4	7.7–8.0	9.0	**0.0**				
7 *N.concinna*	14.8–15.4	12.7	14.5	8.6–9.0	7.7	9.6	**0.0**			
8 *N.radula*	20.1–21.3	16.7–17.6	14.5	9.6–11.1	12.0–12.7	12.0–13.0	14.2–14.5	**0.0–2.2**		
9 *N.gloriosa*	21.6–23.1	21.3–22.5	22.2–23.5	24.4–25.0	23.5–24.1	21.9–22.2	22.2–22.5	25.0–25.9	**0.0–1.2**	
10 *L.kogamogai*	23.8–24.1	23.1	25.3	25.3–25.6	24.7	25.3	25.3	28.1–28.4	24.4–25	**0.0**
16S rRNA										
1 *N.nigrans*	**0.0–0.7**									
2 *N.habei*	9.3–9.5	**0.0**								
3 *N.teramachii*	8.7–9.4	8.9–9.1	**0.0–0.2**							
4 *N.fuscoviridis*	12.6–13.4	14.9–15.2	11.1–11.6	**0–0.2**						
5 *N.boninensis*	11–11.7	14.3–14.3	11.3–11.5	9.3–9.5	**0.0**					
6 *N.schrenckii*	12.8–13.5	13.8–14.3	12.5–13.2	10.7–11.4	8.2–8.4	**0.2–0.3**				
7 *N.concinna*	11.2–12.1	11.7–12	10.4–10.9	9.0–9.5	7.9–8.2	8.0–8.4	**0.0–0.2**			
8 *N.radula*	11.5–12.6	12.7–13.4	11.3–12.3	9.3–9.7	8.7–10.7	8.9–10.7	8.2–9.3	**0.0–2.0**		
9 *N.gloriosa*	26.1–26.4	22.4–22.7	24.3–24.9	28.1–28.5	24.9–25.2	22.3–23.2	26.4–27.0	25.8–26.1	**0.0–0.2**	
10 *L.kogamogai*	25.2–25.5	22.8–22.8	26.2–26.5	29.9–30.0	27.9–27.9	28.8–29.5	27.8–28.1	28.5–29.9	28.8–28.8	**0.0**

### ﻿Molecular phylogenetic analysis

**Figure 2.**
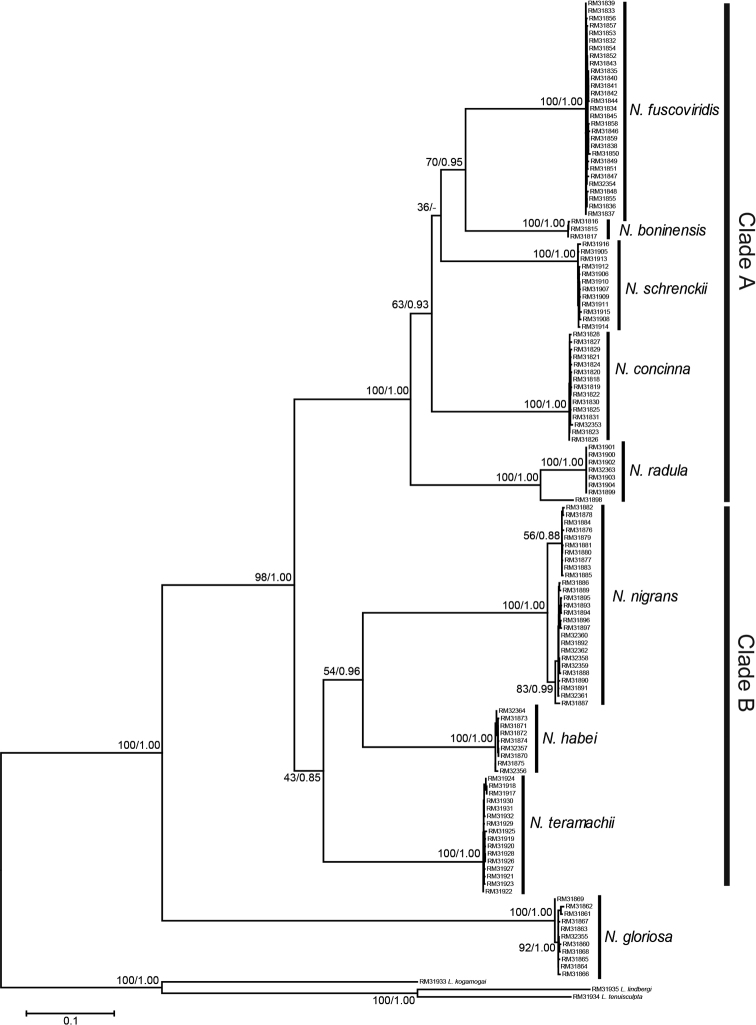
Maximum likelihood phylogenetic tree generated from 1809 bp constructed from the concatenated COI, Cytb, 12S rRNA, and 16S rRNA gene sequences from *Nipponacmea* representatives. Numbers above or below the branches are ML bootstrap values and Bayesian posterior probabilities, respectively. See Table 2 for sample numbers.

The resultant phylogenetic tree using the four genes is shown in Fig. 2. The monophyly of the genus *Nipponacmea* was supported with a bootstrap value (BS) = 100% and posterior probability (PP) = 1.00. There are nine terminal clades, and morphological characters of the sequenced specimens confirmed that these clades corresponded to the *Nipponacmea* species previously defined by Sasaki and Okutani (1993, 1994a) (see below for more notes on the morphology). The relationships among species indicated that: (1) *N.gloriosa* is the sister to the remaining lineages, (2) the remaining species form a large clade supported with BS = 99% and PP = 1.00, and (3) the large clade is divided into two subclades, which we have referred to as Clades A and B. The monophyly of Clade A was well supported with BS = 100% and PP = 1.00. The topology within Clade A was: (*N.radula*, *N.concinna*, *N.schrenckii*, (*N.boninensis*, *N.fuscoviridis*)). BS values for interspecific relationships within this clade were less than 70%, and its branches were not well supported. The highest value within Clade A was between *N.fuscoviridis* and *N.boninensis* (BS = 66%, PP = 0.96). Clade B was supported with BS = 58% and PP = 0.94, and the topology within this group was: (*N.teramachii*, (*N.nigrans*, *N.habei*)). The highest supported values within Clade B were BS = 61% and PP = 0.99 between *N.nigrans* and *N.habei*.

Separate analyses of the four genes resulted in slightly different phylogenetic relationships that are described below. The divergence within *Nipponacmea* in the COI tree (Suppl. material 1: Fig. S1) was expressed as: (Clade A, (Clade B, *N.gloriosa*)), whereas in the tree constructed with combined sequences, *N.gloriosa* was a sister to the other lineages. The topology within Clade A, unlike what was revealed with the combined sequence tree, was: ((*N.fuscoviridis*, *N.concinna*), (*N.schrenckii*, (*N.radula*, *N.boninensis*))), whereas that for Clade B was the same as that of the combined tree. Phylogenetic relationships within *Nipponacmea* species were different from those of the combined tree in the Cytb analysis (Suppl. material 2: Fig. S2). The topology within Clade A was: (*N.boninensis*, (*N.fuscoviridis*, (*N.concinna*, (*N.schrenckii*, *N.radula*)))), while Clade B showed: (*N.teramachii*, (*N.nigrans*, (*N.habei*, *N.gloriosa*))). Relationships among species were similar to those of the combined tree in the analysis of 12S rRNA gene (Suppl. material 3: Fig. S3). The result of phylogenetic analysis of 16S rRNA gene is shown in Suppl. material 4: Fig. S4. As in the combined tree, *N.gloriosa* was the sister to the remaining *Nipponacmea*, Clade A was well supported, and the topology within that clade was the same as that of the tree of combined sequences. In comparison to the combined tree, the monophyly of Clade B was not supported in the analysis of the 16S rRNA.

**Figure 3.**
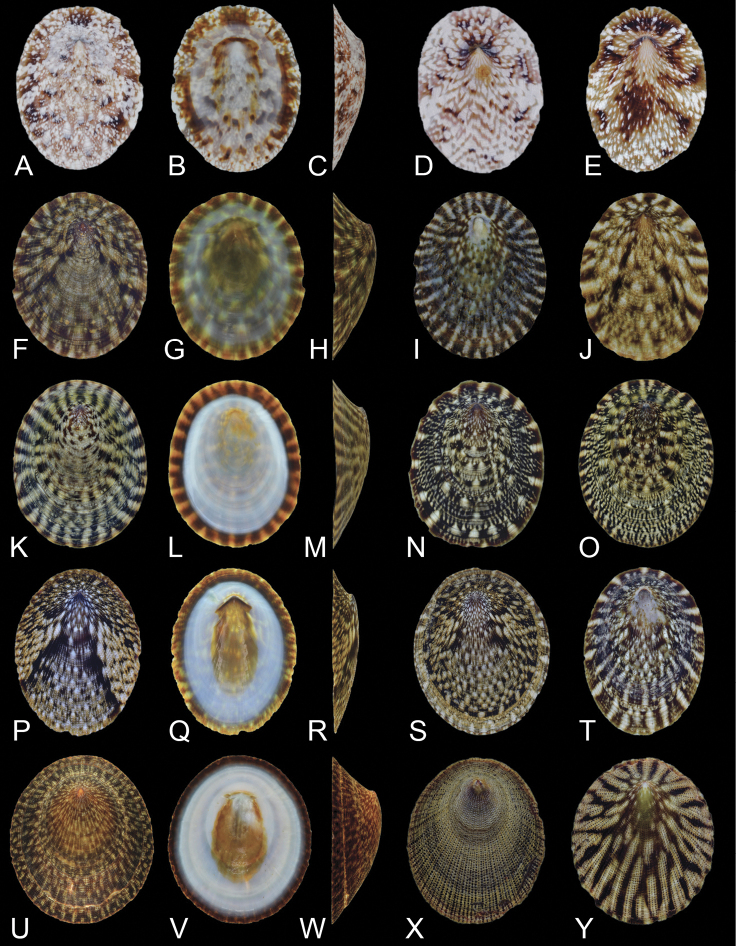
Shell morphology and color pattern of *Nipponacmeagloriosa* and four species of Clade A **A–C***N.gloriosa*, RM31869, Ibusuki, Kagoshima (41) **D***N.gloriosa*, RM31860, Tateyama, Chiba (13) **E***N.gloriosa*, RM31862, Manazuru, Kanagawa (14) **F–H***N.fuscoviridis*, RM31858, Kimotsuki, Kagoshima (42) **I***N.fuscoviridis*, RM31846, Nikaho, Akita (10) **J***N.fuscoviridis*, RM31859, Kimotsuki, Kagoshima (42) **K–M***N.boninensis*, RM31817, Chichijima Is., Ogasawara (43) **N***N.boninensis*, RM31815, Chichijima Is., Ogasawara (43) **O***N.boninensis*, RM31816, Chichijima Is., Ogasawara (43) **P–R***N.schrenckii*, RM31906, Kazamaura, Aomori (6) **S***N.schrenckii*, RM31908, Kazamaura, Aomori (6) **T***N.schrenckii*, RM31916, Nagatamachi, Nagasaki (35) **U–W***N.concinna*, RM31820, Ofunato, Iwate (11) **X***N.concinna*, RM31824, Mihamacho, Wakayama (21) **Y***N.concinna*, RM31828, Suo-Oshima, Yamaguchi (30). Scale bars: 5 mm.

Although the monophyly of Clade A was well supported, branching order within the clade was not (BS values < 70%). In contrast, the monophyly of clade B was not strongly supported, nor was the monophyly of *N.nigrans* and *N.habei* (BS = 54%). Perhaps not surprisingly, separate analyses of the four genes resulted in slightly different trees (Suppl. material 1: Fig. S1, Suppl. material 2: Fig. S2, Suppl. material 3: Fig. S3, Suppl. material 4: Fig. S4).

**Figure 4.**
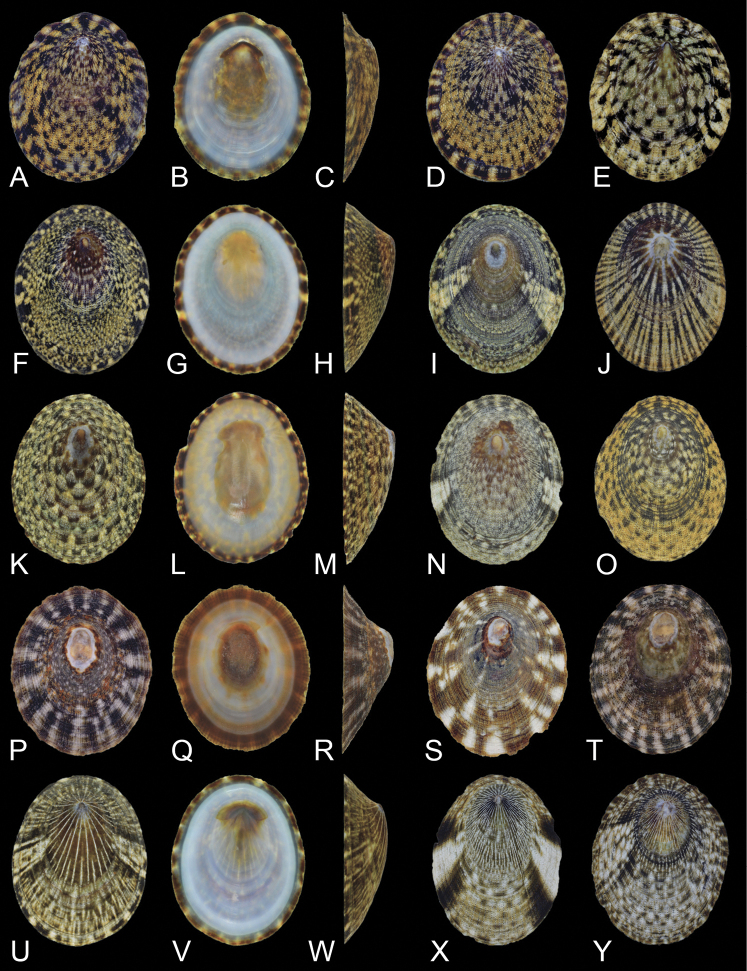
Shell morphology and color pattern of *N.radula* and three species of clade B **A–C***N.radula*, RM31904, Omura, Nagasaki (34) **D***N.radula*, RM31902, Omura, Nagasaki (34) **E***N.radula*, RM31899, Nagato, Yamaguchi (31) **F–H***N.nigrans*, RM31892, Nishiku, Fukuoka (32) **I***N.nigrans*, RM31888, Kada, Wakayama (22) **J***N.nigrans*, RM31895, Higashisonogi, Nagasaki (33) **K–M***N.nigrans*, RM31887, Minamiizu, Shizuoka (15) **N***N.nigrans*, RM31886, Minamiizu, Shizuoka (15) **O***N.nigrans*, RM31897, Higashisonogi, Nagasaki (33) **P–R***N.habei*, RM31874, Ishinomaki, Miyagi (12) **S***N.habei*, RM31875, Tateyama, Chiba (13) **T***N.habei*, RM31873, Usujiri, Hokkaido (5) **U–W***N.teramachii*, RM31930, Nishiku, Fukuoka (32) **X***N.teramachii*, RM31925, Sanuki, Kagawa (28) **Y***N.teramachii*, RM31922, Ainancho, Ehime (24). Scale bars: 5 mm.

### ﻿Morphological characters

In this study, we tested the identification of *Nipponacmea* species based only on sequences, and the results revealed nine phylogenetic groups, which confirmed the nine species currently described. In addition, scientific names were verified by comparison between type and sequenced specimens according to morphological traits. Among numerous possible morphological and anatomical characters, the following six characters were revealed to be most reliable for *Nipponacmea* species identification (Table 5).

**Table 5.** Diagnostic characters of *Nipponacmea* species distributed in Japan.

Species	Shell sculpture		Animal pigmentation			Radula sac	Radular teeth	Ovary
	Granules	Riblets	Snout	Cephalic tentacles	Foot			
*N.gloriosa*	Elongate and thin	Fine and sparse	Non-pigmented	Non-pigmented	Non-pigmented	Short	Blunt	Red
*N.fuscoviridis*	Elongate and thin	Fine and sparse	Non-pigmented	Black	Non-pigmented	Long, posterior and right loops	Acute	Green
*N.boninensis*	Absent	Fine and dense	Non-pigmented	Black	Gray	Intermediate	Slightly blunt	Red
*N.schrenckii*	Elongate and thin	Fine and sparse	Black	Black	Black	Intermediate	Acute	Green
*N.concinna*	Rounded	Absent	Black	Black	Black	Long, posterior and right loops	Acute	Brown
*N.radula*	Pointed	Fine and sparse	Gray	Black	Gray	Long, posterior and right loops	Acute	Brown
*N.nigrans*	Elongate and thcik	Thick and dense	Gray	Black	Gray	Short	Acute	Brown
*N.habei*	Elongate and thin	Fine and dense	Black	Black	Black	Variable from long to short loops	Acute to blunt	Brown
*N.teramachii*	Elongate and thin	Absent	Black	Black	Black	Short	Acute	Brown

**Figure 5.**
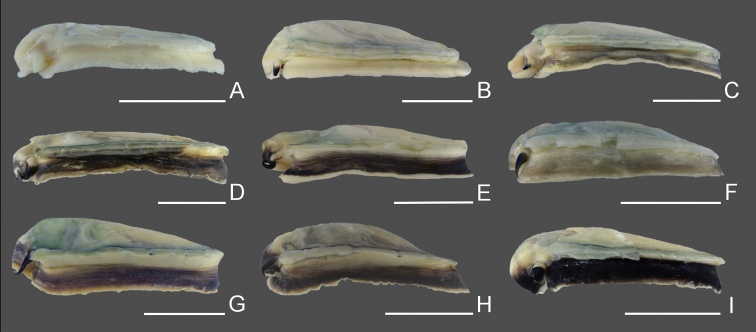
Pigmentation of side of foot **A***N.gloriosa*, RM31861, Manazuru, Kanagawa (14) **B***N.fuscoviridis*, RM31847, Tateyama, Chiba (13) **C***N.boninensis*, RM31816, Chichijima Is., Ogasawara (43) **D***N.schrenckii*, RM31908, Kazamaura, Aomori (6) **E***N.concinna*, RM31830, Omura, Nagasaki (34) **F***N.radula*, RM31900, Nagato, Yamaguchi (31) **G***N.nigrans*, RM32361, Kushimoto, Wakayama (20) **H***N.habei*, RM31870, Otaru, Hokkaido (2) **I***N.teramachii*, RM31917, Tateyama, Chiba (13). Scale bars: 5 mm.

**Figure 6.**
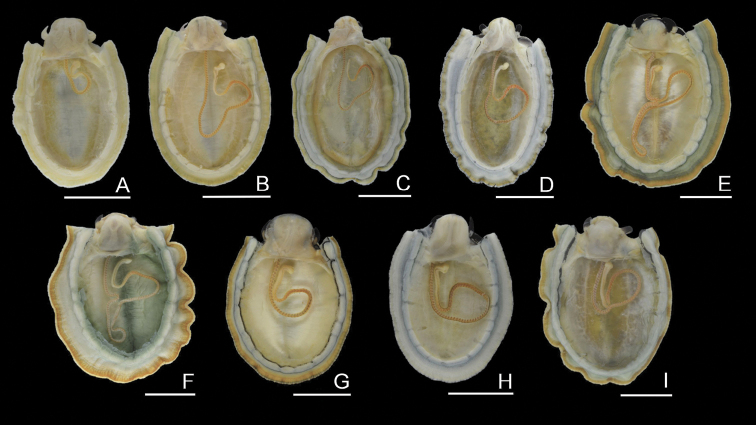
Configuration of radula sac of nine species of *Nipponacmea***A***N.gloriosa*, RM32355, Ibusuki, Kagoshima (41) **B***N.fuscoviridis*, RM32354, Akune, Kagoshima (39) **C***N.boninensis*, RM31817, Chichijima Is., Ogasawara (43) **D***N.schrenckii*, RM31906, Kazamaura, Aomori (6) **E***N.concinna*, RM32353, Nagatamachi, Nagasaki (35) **F***N.radula*, RM32363, Akune, Kagoshima (37) **G***N.nigrans*, RM32362, Kushimoto, Wakayama (20) **H***N.habei*, RM32356, Tateyama, Chiba (13) **I***N.teramachii*, RM31928, Suo-Oshima, Yamaguchi (30). Scale bars: 5 mm.

**Figure 7.**
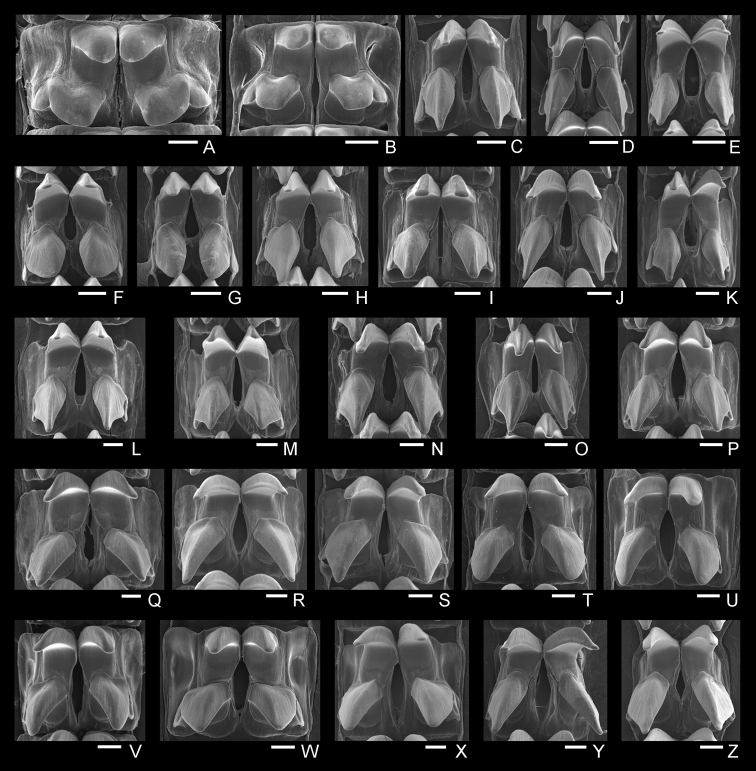
Scanning micrographs of radular teeth of of *Nipponacmea***A***N.gloriosa*, RM32355, Ibusuki, Kagoshima (41) **B***N.gloriosa*, RM31860, Tateyama, Chiba (13) **C***N.fuscoviridis*, RM31858, Kimotsukicho, Kagoshima (42) **D***N.fuscoviridis*, RM32354, Akune, Kagoshima (39) **E***N.fuscoviridis*, RM31834, Rumoi, Hokkaido (1) **F***N.boninensis*, RM31817, Chichijima Is., Ogasawara (43) **G***N.boninensis*, RM31815, Chichijima Is., Ogasawara (43) **H***N.schrenckii*, RM31915, Suo-Oshima, Yamaguchi (30) **I***N.schrenckii*, RM31906, Kazamaura, Aomori (6) **J***N.schrenckii*, RM31916, Nagatamachi, Nagasaki (35) **K***N.concinna*, RM31831, Omura, Nagasaki (34) **L***N.concinna*, RM32353, Nagatamachi, Nagasaki (35) **M***N.concinna*, RM31823, Tahara, Aichi (19) **N***N.radula*, RM31898, Hamamatsu, Shizuoka (18) **O***N.radula*, RM31904, Omura, Nagasaki (34) **P***N.radula*, RM32363, Akune, Kagoshima (37) **Q***N.nigrans*, RM32360, Kushimoto, Wakayama (20) **R***N.nigrans*, RM32359, Kushimoto, Wakayama (20) **S***N.nigrans*, RM32358, Kushimoto, Wakayama (20) **T***N.habei*, RM32364, Tateyama, Chiba (13) **U***N.habei*, RM31872, Suttu, Hokkaido (3) **V***N.habei*, RM31873, Usujiri, Hokkaido (5) **W***N.habei*, RM32357, Usujiri, Hokkaido (5) **X***N.habei*, RM32356, Tateyama, Chiba (13) **Y***N.teramachii*, RM31926, Sanuki, Kagawa (28) **Z***N.teramachii*, RM31924, Ohira, Oita (25). Scale bars: 50 μm.

(1) Granules: Granules on the shell exterior exhibited five character states: (a) rounded (*N.concinna*), (b) pointed (*N.radula*), (c) smooth (*N.boninensis*), (d) thickly elongated (*N.nigrans*), and (e) thinly elongated (the remaining species). These results corroborate previous observations by Sasaki and Okutani (1993; fig. 15). The phylogeny suggests granules were differentiated according to species-specific types in Clade A, such as the elongate type seen in *N.gloriosa*, and Clade B.

(2) Riblets: Exterior riblets were either fine, rough, or absent, depending on species. In Clade A, the riblets were fine and sparse in *N.fuscoviridis*, *N.schrenckii*, *N.radula*, while they were fine and dense in *N.boninensis*, and absent in *N.concinna*. In Clade B, the riblets were thick and dense in *N.nigrans*, fine and dense in *N.habei*, and absent in *N.teramachii*. The topology of the molecular phylogenetic trees indicated that the riblets do not reflect phylogeny.

(3) Animal pigmentation: Pigmentation in the snout, cephalic tentacles, and side of the foot was divergent among species, including black, grey, or non-pigmented types (Fig. 5). The snout was not pigmented in *N.gloriosa*, *N.fuscoviridis*, or *N.boninensis*; lightly pigmented in *N.radula* and *N.nigrans*; and blackened in the remaining four species. The pigmentation of the snout did not reflect phylogenetic relationships. Only *N.gloriosa* lacked pigmentation in the cephalic tentacles, whereas the other eight species had darkly pigmented tentacles. The side of the foot was not pigmented in *N.gloriosa* or *N.fuscoviridis*, lightly pigmented in *N.boninensis*, *N.radula*, and *N.nigrans*, and finally darkly pigmented in the remaining four species. Relationships between pigmentation patterns and phylogeny were not detected.

(4) Radular sac: The configuration of the radular sac was different among the species (Fig. 6). *Nipponacmeaconcinna* and *N.radula* had two loops, the anterior and posterior loops, while the other species formed a single shorter loop. Again, this character did not correspond with the defined phylogenetic relationships.

(5) Radular teeth: The lateral teeth were short and blunt in *N.gloriosa*, long and slightly blunt in *N.boninensis*, and long and acute in the rest of the species (Fig. 7). The radular morphology of *N.habei* teeth showed a wider range of variation than that of the remaining species in regard to the acuteness of the middle lateral teeth.

(6) Ovary: The color of the ovary can be classified into three categories: green in *N.fuscoviridis* and *N.schrenckii*, red in *N.boninensis* and *N.gloriosa*, and brown in *N.concinna*, *N.radula*, *N.teramachii*, *N.nigrans*, and *N.habei*. The ovaries of all species in Clade B were pigmented brown, whereas those of Clade A were variable and are characterized by one of the three color patterns outlined above.

### ﻿Morphometric analysis

**Figure 8.**
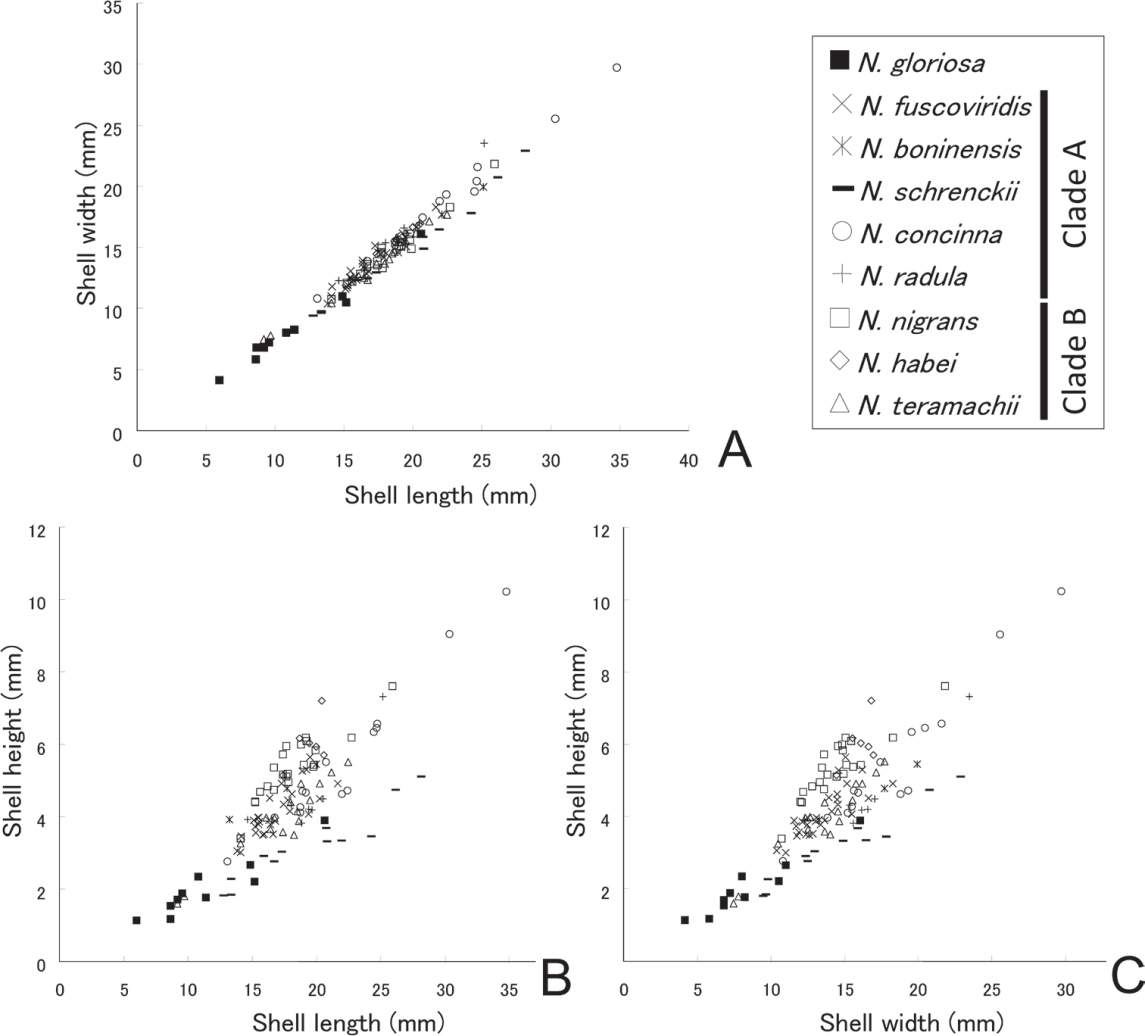
Relationships among shell length, width, and height.

**Figure 9.**
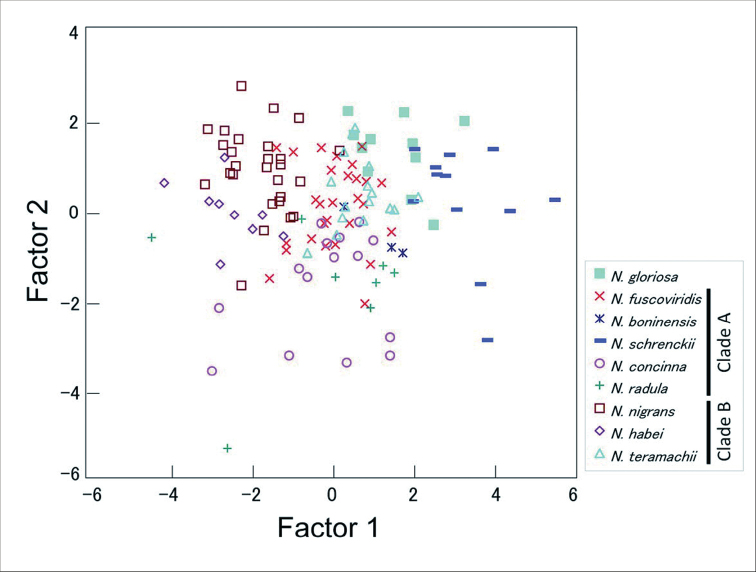
Plot of the results of discriminant function analysis of shell length, width, and height for individuals of *Nipponacmea* species.

The relationships among length, width, and height are indicated in Fig. 8 and were similar among species; however, the correlations between length and height, and between width and height differed. The results of Welch’s t-test using the proportion of length and height indicated that the apex height of Clade B (average H/L ratio = 0.27) was significantly higher than that of Clade A (average H/L ratio = 0.22; t = 5.24, P = 0.001). Applying the canonical discriminant analysis, only 51.9% of the original 130 individuals were assigned to the correct species (Fig. 9, Table 6). Therefore, it is difficult to distinguish between the nine genetic species solely from shell morphometry. *Nipponacmeanigrans* was discriminated best, with 23 out of 27 correctly matched individuals, while *N.boninensis* was the least discriminated, with 0 out of 3 individuals classified correctly.

**Table 6.** Canonical discriminant analysis for individuals of *Nipponacmea* species identified with mtDNA sequences.

Observed classification	Predicted classification									
	1	2	3	4	5	6	7	8	9	% correct
1 *N.gloriosa*	8	0	0	3	0	0	0	0	0	72.7
2 *N.fuscoviridis*	0	23	0	0	1	1	2	0	2	79.3
3 *N.boninensis*	0	1	0	1	0	0	0	0	1	0.0
4 *N.schrenckii*	3	0	0	8	0	0	0	0	1	66.7
5 *N.concinna*	0	7	0	0	7	0	0	1	0	46.7
6 *N.radula*	0	4	0	0	2	1	0	1	0	12.5
7 *N.nigrans*	0	3	0	0	0	0	23	1	0	85.2
8 *N.habei*	0	1	0	0	0	0	6	2	0	66.7
9 *N.teramachii*	0	9	0	1	0	0	0	0	6	37.5

## ﻿Discussion

### ﻿Monophyly of species

The monophyly of Japanese *Nipponacmea* species has not been previously tested using molecular characters; however, it was strongly supported by the data obtained from the present study (Fig. 2). The taxonomy of patellogastropod species based on morphological characters can be frustrated due to polyphenism (*Patelloida*: Nakano and Ozawa 2005, *Notoacmeascapha*: Nakano and Spencer 2007; Nakano et al. 2009a) or the existence of cryptic species (*Notoacmea* species: Nakano and Spencer 2007; Nakano et al. 2009a, *Nacella* species; de Aranzamendi et al. 2009; González-Wevar et al. 2011). In the present study, neither polyphenism nor cryptic species were found in *Nipponacmea*.

**Table 7.** Holotype specimens, type localities, and geographic distribution of *Nipponacmea* species.

Species	Holotype	Type locality	Geographic distribution
*N.gloriosa* (Habe, 1944)	National Museum of Nature and Science,Tsukuba, NSMT-Mo 100675	Urado, Kochi Prefecture	Pacific coast from Choshi to Kyushu, the Sea of Japan from Oga Peninsula to Kyushu, and rare in Seto Inland Sea; China.
*N.fuscoviridis* (Teramachi, 1949)	Teramachi Collection in Toba Aquarium, missing	Akune, Kagoshima Prefecture	Pacific coast and the Sea of Japan from southern Hokkaido to Kyushu, and Ryukyu Islands; Korea, China.
*N.boninensis* (Asakura & Nishihama, 1987)	National Museum of Nature and Science,Tsukuba, NSMT-Mo 64445	Yagyu-san, Chichijima Island, Ogasawara Islands	Hachijo Island, Ogasawara Islands, and Northern Mariana Islands (Asuncion and Maug Islands)
*N.schrenckii* (Lischke, 1868)	Unknown	Nagasaki City	Tsugaru Strait to Kyushu, and Seto Inland Sea; Korea, China.
*N.concinna* (Lischke, 1870)	Unknown	Nagasaki City	Pacific coast and the Sea of Japan from Hokkaido to Kyushu, and Seto Inland Sea; Korea.
*N.radula* (Kira, 1961)	Osaka Museum of Natural History, Kira Collection 525	Akune, Kagoshima Prefecture	Pacific coast from Shizuoka Prefecture to Kyushu, the Sea of Japan from Yamaguchi Prefecture to Kyushu, and Seto Island Sea; Korea, China.
*N.nigrans* (Kira, 1961)	Osaka Museum of Natural History, Kira Collection 540	Shionomisaki, Kii Peninsula	Pacific coast and the Sea of Japan from Hokkaido to Kyushu, and Seto Inland Sea; Korea, China, Taiwan.
*N.habei* Sasaki & Okutani, 1994	National Museum of Nature and Science,Tsukuba, NSMT-Mo 69985	Shiragami-misaki, Matsumae, Hokkaido	Pacific coast from Hokkaido to Izu Peninsula, the Sea of Japan from Hokkaido to Niigata Prefecutre
*N.teramachii* (Kira, 1961)	Osaka Museum of Natural History, Kira Collection 554	Akune, Kagoshima Prefecture	Pacific coast from Ojika Peninsula to Kyushu, western and northern Kyushu, and Seto Inland Sea; Korea, China.
*N.formosa* (Christiaens, 1977)	Natural History Museum, London, No. 1977167	Northern Taiwan	Taiwan
*N.vietnamensis* Chernyshev, 2008	Zoological Museum of Far East State University, No. 18852	Gulf of Tonkin	Vietnam
*N.moskalevi* Chernyshev & Chernova, 2002	Zoological Museum of Far East State University, No H 2666	Japan Sea, Sukhoputnaya Bay	Far East Russia

In this study, the maximum genetic distance within species was noticeably smaller than the minimum among species; therefore, the genetic distances were consistent with morphology-based species taxonomy. The maximum genetic distance within Japanese *Nipponacmea* species was 9.9% in COI in *N.radula* (Table 4). The minimum genetic distance was 17.8% in COI between *N.boninensis* and *N.schrenckii*. The genetic distances among species in *Notoacmea* in New Zealand ranged from 3.94% to 44.4% for COI, and distances within species were from 0.00% to 2.96% (Nakano and Spencer 2007; Nakano et al. 2009a). Thus, genetic distances are greatly variable among species in the New Zealand *Notoacmea* and the Japanese *Nipponacmea*.

A comparison of holotype and sequenced specimens from type localities (topotypes) is useful to confirm species identity. We investigated holotypes of seven species (*N.radula*, *N.boninensis*, *N.habei*, *N.teramachii*, *N.nigrans*, *N.gloriosa*, and *N.formosa*), excluding *N.schrenckii*, *N.concinna*, and *N.fuscoviridis* whose type materials are currently missing (Table 6). Morphological comparisons between sequenced specimens and holotypes were possible when considering characters related to shell surface sculpture (riblets and granules). In addition, sequence data of topotypes are important to precisely identify sequenced specimens. In this study, genetic variation was not significant among individuals of the four species collected from their type localities (*N.boninensis*, *N.fuscoviridis*, *N.nigrans*, and *N.teramachii*). The maximum genetic distances among COI sequences of topotypes of these species were 0.4% for *N.boninensis*, and 0.2% for *N.fuscoviridis*, *N.nigrans*, and *N.teramachii*. Thus, the molecular phylogeny corroborated the morphology-based taxonomy originally defined in the 1990s.

### ﻿Phylogenetic relationships among *Nipponacmea* species

The results of the molecular phylogenetic analysis in this study revealed three major clades (*N.gloriosa*, Clade A, and Clade B), with *N.gloriosa* as sister to the other *Nipponacmea* species. This relationship is consistent with delineations observed based on major differences observed in radular morphology, food preference, and habitat. *Nipponacmeagloriosa* grazes exclusively on coralline algae, while the other species consume different materials, for example, *N.concinna* is known to graze on *Ulva* spp. (Kawakami and Habe 1986). Additionally, *N.gloriosa* is the only species that inhabits the subtidal zone; the others are restricted to the intertidal zone (Sasaki and Okutani 1993; Sasaki 2000, 2017).

Clade A was robustly supported with high bootstrap values by Nakano and Ozawa (2007) (BS = 99%) as well as in this study (BS = 100%). Branching order within the clade is as follows: *N.radula*, *N.concinna*, *N.schrenckii*, and *N.fuscoviridis*, with the latter as the most derived species in this clade. *Nipponacmeaboninensis* was recently included in the phylogenetic analysis in this study and formed a clade with *N.fuscoviridis*. Asakura and Nishihama (1987) compared *N.boninensis* to *N.schrenckii*, but Nakano (2007) mentioned similarities between *N.boninensis* and *N.fuscoviridis* regarding morphological and ecological characters. In this study, the latter hypothesis was clearly supported.

The monophyly of Clade B was supported with relatively lower bootstrap values than that of Clade A (BS = 80% by Nakano and Ozawa (2007); and BS = 67% in this study). Phylogenetic relationships within Clade B were inconstant among different analyses. In this study, *N.teramachii* diverges first, and *N.nigrans* and *N.habei* are more closely related (BS = 75%). Previous studies revealed that *N.nigrans* is separated first, and *N.habei* and *N.teramachii* form a clade (BS = 80%) (Nakano and Ozawa 2007).

Differences exist in the aims and taxa sampled between our studies and previous research focused on *Nipponacmea*; however, the results are not contradictory. Compared to previous studies, we improved the phylogenetic analyses and validation of species taxonomy and taxonomic characters by: (1) obtaining novel sequence data from *N.boninensis* for the first time; (2) using the most diverse taxon sampling for *Nipponacmea* to date, including multiple specimens (ranging from 3 to 29) for each species, for a total of 130 specimens from 43 localities and 9 species; and (3) obtaining sequence data for Cytb in addition to other three mitochondrial (COI, 12S, and 16S rRNA) genes. The Cytb gene was used in this study since it evolves at higher rates than the 16S and is better for investigation of among-species and among-populations relationships.

### ﻿*Nipponacmea* species taxonomy

The species taxonomy of *Nipponacmea* had long been confused prior to revision by Sasaki and Okutani (1993). The chief cause of this confusion and misidentification was an overemphasis of the importance of shell color pattern. Four to seven species occur sympatrically in temperate Japanese waters, and the distinction and taxonomic rank of these species or subspecies has been contested by various authors (see Sasaki and Okutani 1993 for details). A similar situation also existed in the New Zealand genus *Notoacmea*, before a phylogenetic analysis and taxonomic revision of this genus was performed by Nakano and Spencer (2007) and Nakano et al. (2009) reporting cryptic species and phenotypic polymorphisms. These anomalies were not found in the present study with *Nipponacmea*, and the DNA-based clades were consistent with the morphological species recognized by Sasaki and Okutani (1993). Based on the results of phylogenetic analysis, we discuss the validity and current issues concerning the definition of each species below.

(1) *Nipponacmeagloriosa*: *N.gloriosa* is the exclusive species living in the subtidal zone that grazes on coralline algae (Sasaki and Okutani 1993; Sasaki 2000, 2017). This species was originally described based on shell morphology, shell color, and radula (Habe 1944). The shell is reddish, while the head, cephalic tentacles, and side of the foot are not pigmented (Table 5). Juveniles of *N.gloriosa* can be easily distinguished from those of other *Nipponacmea* species by their reddish-brown radial lines (Sasaki 2006). On morphological grounds, Sasaki and Okutani (1994b) regarded *Collisellacellanica* from Hong Kong as a junior synonym of *N.gloriosa*; this species should be investigated using molecular phylogenetic analysis in the future. It is unclear whether *N.gloriosa* is present outside of Japan in places such as South Korea or Taiwan.

(2) *Nipponacmeafuscoviridis*: The holotype of *N.fuscoviridis* (Teramachi, 1949) was apparently held in the Toba Aquarium’s Teramachi Collection, but its location cannot be confirmed. Currently, the identity of this species is based on the topotype specimens collected by Teramachi and preserved in the Kira Collection (Sasaki et al. 2014). For an unclear reason *N.fuscoviridis* was previously regarded as a subspecies of *N.concinna* (Kira 1954; Habe and Kosuge 1967; Kuroda et al. 1971; Okutani and Habe 1975). *Nipponacmeafuscoviridis* is the only species of the genus found in the Ryukyu Islands (Sasaki and Okutani 1993; Sasaki and Nakano 2007), and it is also distributed in South Korea (Min 2001; Noseworthy et al. 2007) and China (Yu et al. 2014).

Two morphologically similar species are known from Taiwan and Vietnam. Christiaens (1980) described *Collisellaformosa* from northern Taiwan based on shell and radula morphology, and Sasaki and Okutani (1994b) suggested that *C.formosa* belongs to *Nipponacmea*. We examined the holotype specimen and concluded that *N.formosa* is most similar to *N.fuscoviridis* based on color pattern and features of the shell sculpture. The validity of *N.formosa* should be verified by molecular characters in future studies. Chernyshev (2008) described *N.vietnamensis* from the Gulf of Tonkin, located in northern Vietnam. *Nipponacmeavietnamensis* is very similar to *N.fuscoviridis*, but it has a different shell color and a characteristic reddish ovary (Chernyshev 2008). The distribution of *N.formosa* and *N.vietnamensis* is geographically separate, but similarity in morphological features suggest they are phylogenetically close and, therefore, these species should also be compared using molecular makers.

(3) *Nipponacmeaboninensis*: In the original description, *N.boninensis* was compared to *N.schrenckii* based on shell and radula morphology (Asakura and Nishihama 1987). However, Nakano (2007) highlighted that *N.boninensis* is more similar to *N.fuscoviridis* based on shell color patterns and habitat. In this study, we confirmed that *N.boninensis* is more closely related to *N.fuscoviridis* than *N.schrenckii* genetically. Morphologically this relationship is supported by the outline, apex height, and color pattern of the shell, as well as the pigmentation on the side of the foot, and arrangement of the radular sac (Table 5). The genetic distances indicate that *N.boninensis* is closely related to *N.fuscoviridis* according to the Cytb and 12S rRNA genes (17.1% and 5.6%, respectively). Therefore, *N.boninensis* is clearly differentiated from the other species morphologically and genetically, and should be regarded as an independent species.

*Nipponacmeaboninensis* is an endemic species to the southern Izu Islands (Hachijo Island), Ogasawara Islands, and the northernmost part of the Northern Mariana Islands (Asuncion and Maug Islands: Asakura and Kurozumi 1991: figs 1–3). There are no other *Nipponacmea* species recorded in the Izu-Ogasawara Islands or southward of this region. Fukuda (1993, 1994, 1995a, b) stated that temperate mollusks in the Ogasawara Islands are conveyed by Kuroshio currents from southern Honshu. In the genus *Cellana*, ancestral species possibly reached the Ogasawara Islands through the Izu Islands as stepping-stones (Nakano et al. 2009b). Similar to *Cellana*, the ancestral species of *N.boninensis* was assumed to have migrated from Honshu to the Ogasawara Islands through the Izu Islands.

(4) *Nipponacmeaschrenckii*: *N.schrenckii* has the lowest shell apex among *Nipponacmea* species (Takada 1992). Lischke’s (1868) holotype is apparently lost, but illustrations from the original literature are clear, leading to few challenges concerning the taxonomic status of the species (Table 6; Lischke 1869). *Nipponacmeaschrenckii* also occurs in South Korea (Noseworthy et al. 2007) and China (Huang 2008; Liu 2008), but not in Taiwan.

(5) *Nipponacmeaconcinna*: Lischke’s (1870) type is also missing; however, we used the original illustration for identification purposes. Similar to examples of distinct color polymorphism in patellogastropods (Sasaki 1999a, b; Lindberg 2008; Nakano et al. 2010), *N.concinna* has two color forms (solid and spotted) with occasional intermediate variations (Fig. 3U–Y; Sasaki and Okutani 1993; Sasaki 2000, 2017). The results of this study revealed that these two morphs are intermingled in a single clade; thus, the color forms were proven to be intraspecific variations. The spotted form of *N.concinna* and *N.radula* are the most readily confused phenotypes; however, *N.concinna* can be distinguished by rounded granules and black pigmentation in the snout and the side of the foot. The presence of *N.concinna* outside of Japan and in South Korea has been confirmed (Min 2001; Noseworthy et al. 2007); however, no specimens have been found in China or Taiwan.

(6) *Nipponacmearadula*: The distribution of *N.radula* is limited to the southwest area of Japan, which is a small area compared to that of other *Nipponacmea* species. However, intraspecific genetic divergence is high for this genus. *Nipponacmearadula* tends to prefer sheltered environments, and its distribution areas are often isolated. This specialized habitat may lead to the large genetic distances across the entire geographic range of *N.radula* (within species 9.9% for COI: Table 4). Populations with large genetic distances are completely indistinguishable according to morphological features. The shell height for *N.radula* is relatively low for the genus, and the color pattern is considerably variable (Fig. 4A–E). In the past, this species was misidentified as *N.concinna* or regarded as a subspecies of *N.concinna* (Habe and Kosuge 1967; Nakamura 1986; Takada 1992). *Nipponacmearadula* was found outside of Japan, in South Korea (Min 2001; Noseworthy et al. 2007) and China (Yu et al. 2014), but not in Taiwan.

(7) *Nipponacmeanigrans*: The shell height of *N.nigrans* is relatively high, and the color patterns and shell shape are highly variable (Fig. 3K–T). The individuals from northeastern Japan are more darkly colored, whereas southwestern Japanese populations are lighter. Like *N.radula*, *N.nigrans* has been confused with *N.concinna* (or regarded as a subspecies of *N.concinna*) (Habe and Kosuge 1967; Kuroda et al. 1971; Nakamura 1986). *Collisellamortoni*, Christiaens, 1980 is possibly a junior synonym of this species (Sasaki & Okutani, 1994b). Another similar-looking species, *N.moskalevi* Chernyshev & Chernova, 2002 was described from Sukhoputnaya Bay, Russia based on differences in the sculpture of shell surfaces. In this species, arrangement of the radular sac and radula morphology is similar to that of *N.nigrans*. Relationships among *N.nigrans* and *N.moskalevi* should be tested using molecular makers in future studies. *Nipponacmeanigrans* also occurs in South Korea (Min 2001), China (Christiaens 1980; Yu et al. 2014), and Taiwan (Teruya pers. obs.).

(8) *Nipponacmeahabei*: This species is distributed mainly in the cold-water region from the Izu Peninsula to southern Hokkaido on the Pacific coast and from Niigata Prefecture to southern Hokkaido in the Sea of Japan (Sasaki and Okutani 1994a; Sasaki 2000, 2017). *Nipponacmeahabei* can be distinguished by its high shell-apex, the lack of a greenish hue inside of the shell, and dark pigmentation.

The arrangement of the radular sac and the morphology of the lateral teeth are more variable in *N.habei* than in other *Nipponacmea* species (Sasaki and Okutani 1994a), and molecular analysis confirmed that the variants belong to the same clade. The lateral teeth have two main forms (blunt and acute), but can also have an intermediate morphology. Sasaki and Okutani (1994a) presumed that the geographic distribution of the two radular forms is controlled by oceanic currents and different food biota, and a similar case was reported in *Notoacmeascapha* in New Zealand (Nakano and Spencer 2007; Nakano et al. 2009a). However, here we could not sufficiently test the hypothesis using molecular phylogenetic analyses due to the small number of localities and sequenced specimens (Fig. 2, Suppl. material 1: Fig. S1, Suppl. material 2: Fig. S2, Suppl. material 3: Fig. S3). Population genetic structure and morphological tendency should be examined in more detail in the future. *Nipponacmeahabei* has not yet been found outside of Japan.

(9) *Nipponacmeateramachii*: Although the name of this species was originally proposed for a form with white radial rays, the shell color pattern of *N.teramachii* is highly variable (Fig. 4). Interestingly, *N.teramachii* juveniles are unexceptionally striated with white radial rays, and most individuals abruptly change their color pattern during ontogeny. According to this juvenile character, *N.teramachii* can easily be distinguished from other *Nipponacmea* species (Sasaki and Okutani 1993; Sasaki 2000, 2017). The variants of *N.nigrans* (e.g., Fig. 4J) with radial rays are similar to *N.teramachii*, but such specimens can be distinguished by the granules on the exterior shell surface. The habitat of *N.teramachii* is limited to slightly sheltered environments. The presence of *N.teramachii* outside of Japan was confirmed in South Korea (Noseworthy et al. 2007), China (Yu et al. 2014), but not in Taiwan.

### ﻿Validity of morphological characters

Morphology-based studies of patellogastropods have explored various animal characteristics (Lindberg 1981, 1988; Sasaki and Okutani 1993; Ridgway et al. 1998; Sasaki 1998) in addition to the basics of shells and radulae (Pilsbry 1891; Suter 1904; Oliver 1926; Thiele 1929; Powell 1973; Ponder and Creese 1980). Comparison with molecular phylogeny confirmed the utility of shell and soft-part characters in *Nipponacmea*, as discussed below.

(1) Shell color pattern: the degree of variability in the shell color pattern is different among species, and the patterns are categorized into three types: (i) striking variations (*N.radula*, *N.habei*, *N.nigrans*, and *N.teramachii*), (ii) faint variations (*N.schrenckii*, *N.gloriosa*, *N.boninensis*, and *N.fuscoviridis*), and (iii) dimorphisms of solid or spotted patterns (*N.concinna*). In *N.concinna*, the distribution of color forms has a geographic bias maintained by unknown factors: the solid type is common to northeastern Japan, while the spotted type is frequently found in southwestern Japan. Northern individuals of *N.nigrans* and *N.habei* also tend to have dark colored shells. Another similar example is the Japanese mud snail, *Batillariaattramentaria*, which exhibits a shell color polymorphism in which darker morphs are distributed in colder regions and lighter morphs are more commonly found in warmer regions (Miura et al. 2007). The authors suggested that shell color polymorphism is caused by climatic selection, which could be the case for the shell color patterns of *N.concinna*, *N.nigrans*, and *N.habei*.

The shell of *N.gloriosa* is reddish brown and completely different from other *Nipponacmea* species (Fig. 3A–E). Patellogastropod species associated with coralline algae in the subtidal zone are generally known to have reddish or white shells (e.g., *Niveotecturapallida*, *Tecturaemydia*, and *Erginussybariticus*; Lindberg 2008), and *N.gloriosa* appears to follow this trend. In this case, the color of the shell might be derived from the pigment of the grazed algae.

(2) Shell sculpture: concerning shell sculpture, ribs and granules on the shell exterior are differentiated among species (Table 5). In multiple limpet groups, species living in sun-exposed rocky surfaces tend to have more prominent sculptures than those in shaded habitats (Vermeij 1973). However, this is not observed in *Nipponacmea* species. For instance, *N.fuscoviridis* is attached to the exposed surface during the highest tidal level, but has a delicately sculptured shell, while *N.nigrans* has the most remarkably ornamented ribs and granules, but prefers relatively sheltered environments, and *N.concinna* has notable granules, but is nocturnal and prefers shaded areas in the daytime (Sasaki pers. obs.). Hence, we cannot detect any fixed ecological pattern linked to microscopic shell sculpture within *Nipponacmea*.

(3) Apex height: Takada (1992) indicated quantitatively that there are variations in height among *Nipponacmea* species. For example, in the ratio of shell length to height, *N.schrenckii* has the lowest apex and *N.nigrans* had the highest among *Nipponacmea* species (fig. 2 in Takada 1992). Japanese species are separated into two groups: *N.gloriosa* and Clade A constitute the low-apex group, and Clade B comprises the high-apex one.

In *Nipponacmea*, the shell height is not relevant to the vertical distribution (Sasaki and Okutani 1993: fig. 28) in the tidal zone. It was previously assumed that variation in limpet apex height is correlated with habitat tidal level (Ino 1935; Vermeij 1973), whereby species with a higher shell apex are assumed to store a larger amount of seawater, which might be an adaptation to prevent desiccation (Vermeij 1973; Branch 1975). In this study, we confirmed that the shell height among *Nipponacmea* species is not correlated with tidal level distributions in the intertidal zone.

The topology of the phylogenetic tree implies that the high-apex group could be derived from the low-apex species, since the most basal species, *N.gloriosa*, and Clade A share a low apex. In the genus *Notoacmea* in New Zealand, 13 species formed two major clades; however, they were not based on shell height (Nakano et al. 2009a). Similarly, in the phylogeny of 15 *Nacella* species, shell height is not correlated with phylogeny (González-Wevar et al. 2011). Thus, shell height in general is not controlled by phylogeny in patellogastropod limpets (Nakano and Sasaki 2011).

(4) Animal pigmentation: we confirmed that the pigmentation of the snout, cephalic tentacle, and side of the foot is different among species (Fig. 5). The side of the foot of three species included in Clade B and *N.schrenckii* of Clade A tends to be pigmented in black. Ecologically, the dark pigmentation on the foot wall might be effective to avoid visible detection by predators. However, actual ecological significance is uncertain regarding the species-specific animal pigmentation patterns in *Nipponacmea*.

*Nipponacmeagloriosa*, which inhabits the subtidal zone, lacks pigmentation, and the pale coloration of this animal is possibly a consequence of its habitat. The limpets inhabiting the subtidal zone are unexceptionally pale (e.g., *Niveotecturapallida*, *Tecturaemydia*, and *Erginussybariticus*; Lindberg 2008). For species that inhabit the range from the middle to upper intertidal zone, animal pigmentation is unrelated to tidal level preference in *Nipponacmea*. For example, both *N.concinna* and *N.fuscoviridis* prefer higher tidal levels, but the former species is darkly pigmented, while the latter lacks pigmentation. Thus, it is not straightforward to correlate animal pigmentation patterns and habitats.

(5) Radular sac: the configuration of the radular sac has been regarded as a useful character for identification of *Nipponacmea* species (Sasaki and Okutani 1993; Sasaki 1999a, b). The looping of this pouch is categorized into four types: (i) a short single loop (*N.gloriosa*), (ii) an intermediate length loop (*N.schrenckii*, *N.boninensis*, *N.nigrans*, and *N.teramachii*), (iii) a long radular sac with two loops (*N.concinna*, *N.fuscoviridis*, and *N.radula*), and finally (iv) a variable type ranging from long to short loops (*N.habei*) (Sasaki and Okutani 1993). In addition to differences among species, vertical distribution in the intertidal zone appears to correlate with radular sac length in *Nipponacmea*, whereby the lengths are longer in species inhabiting the higher intertidal zone and shorter in those in the lower intertidal zone.

(6) Radula: the radula morphology is useful for classifying patellogastropod species (Habe 1944; Macpherson 1955; Moskalev 1970; Ponder and Creese 1980; Lindberg 1981; Lindberg and McLean 1981; Sasaki and Okutani 1993). Clarifying the relationship between food and the radula is important for understanding radula morphology (Lindberg 1988). Among *Nipponacmea* species, *N.concinna* is known to graze on green algae (*Ulva* spp.) (Kawakami and Habe 1986), and *N.gloriosa* is a specialist grazer on coralline algae. The limpets gazing on coralline algae tend to have blunt radula (e.g., *Niveotecturapallida* and *Patelloidasignatoides*), whereas the other *Nipponacmea* species are more likely to reveal acute radulae; however, the teeth of *N.boninensis* and *N.habei* are slightly blunt for an unknown reason. At present, the relationship between radular teeth morphology and feeding habits is unclear for non-coralline algae grazers, since there is a lack of detailed data concerning their feeding preferences.

(7) Ovary: the ovaries of *Nipponacmea* species were categorized into three types: (i) green (*N.fuscoviridis* and *N.schrenckii*); (ii) red (*N.boninensis* and *N.gloriosa*); or (iii) brown (*N.concinna*, *N.radula*, *N.teramachii*, *N.nigrans*, and *N.habei*). In relation to the phylogeny, the ovaries of all species in Clade B are pigmented brown, whereas those of Clade A are variable.

In gastropods, the color of the ovary might be constrained according to taxonomic group (e.g., green in vetigastropods such as *Haliotis* and *Turbo*). However, the ovaries of patellogastropods have diversified into various colors. For example, the ovary is brown in *Patelloidalanx* and green in its congener *P.conulus* (Sasaki pers. obs.). The cause for ovary diversification and the ecological significance of color differences in the Patellogastropoda is unknown.

## ﻿Future studies

In this study, we confirmed that current species identified of the Japanese *Nipponacmea* are corroborated by the results from molecular phylogenetic analyses including topotype sequence data, comparative anatomy, and the reinvestigation of type specimens. This study represents an important step towards the revision of the entire group of Asian *Nipponacmea*. Currently, studying Japanese species is important for two reasons: (1) 9 of 12 nominal species in the genus have been described from Japan, and (2) all Japanese species have older species names and nomenclatural priority over more recently described non-Japanese species. *Nipponacmeaformosa* in Taiwan, *N.vietnamensis* in Vietnam, and *N.moskalevi* in Russia must be verified according to morphology, molecular phylogeny, and ecological traits in future studies. In conclusion, a more comprehensive reinvestigation of the genus *Nipponacmea* must be undertaken using taxonomic, phylogenetic, and phylogeographic analyses over a wide geographic range covering Japan, Korea, Russian Far East, China, Taiwan, and Vietnam.

## Supplementary materials

Supplementary material 1 Figure S1 Data type Phylogenetic tree Explanation note Fig. S1. Maximum likelihood phylogenetic tree of COI. Numbers above or below the branches are ML bootstrap and Bayesian posterior probabilities, respectively. https://binary.pensoft.net/file/650860This dataset is made available under the Open Database License (http://opendatacommons.org/licenses/odbl/1.0/). The Open Database License (ODbL) is a license agreement intended to allow users to freely share, modify, and use this Dataset while maintaining this same freedom for others, provided that the original source and author(s) are credited.Shinnosuke Teruya, Davin H. E. Setiamarga, Tomoyuki Nakano, Takenori Sasaki

Supplementary material 2 Figure S2 Data type Phylogenetic tree Explanation note Fig. S2. Maximum likelihood phylogenetic tree of Cytb. Numbers above or below the branches are ML bootstrap and Bayesian posterior probabilities, respectively. https://binary.pensoft.net/file/650861This dataset is made available under the Open Database License (http://opendatacommons.org/licenses/odbl/1.0/). The Open Database License (ODbL) is a license agreement intended to allow users to freely share, modify, and use this Dataset while maintaining this same freedom for others, provided that the original source and author(s) are credited.Shinnosuke Teruya, Davin H. E. Setiamarga, Tomoyuki Nakano, Takenori Sasaki

Supplementary material 3 Figure S3 Data type Phylogenetic tree Explanation note Fig. S3. Maximum likelihood phylogenetic tree of 12S rRNA. Numbers above or below the branches are ML bootstrap and Bayesian posterior probabilities, respectively. https://binary.pensoft.net/file/650862This dataset is made available under the Open Database License (http://opendatacommons.org/licenses/odbl/1.0/). The Open Database License (ODbL) is a license agreement intended to allow users to freely share, modify, and use this Dataset while maintaining this same freedom for others, provided that the original source and author(s) are credited.Shinnosuke Teruya, Davin H. E. Setiamarga, Tomoyuki Nakano, Takenori Sasaki

Supplementary material 4 Figure S4 Data type Phylogenetic tree Explanation note Fig. S4. Maximum likelihood phylogenetic tree of 16S rRNA. Numbers above or below the branches are ML bootstrap and Bayesian posterior probabilities, respectively. https://binary.pensoft.net/file/650863This dataset is made available under the Open Database License (http://opendatacommons.org/licenses/odbl/1.0/). The Open Database License (ODbL) is a license agreement intended to allow users to freely share, modify, and use this Dataset while maintaining this same freedom for others, provided that the original source and author(s) are credited.Shinnosuke Teruya, Davin H. E. Setiamarga, Tomoyuki Nakano, Takenori Sasaki
