# Supplementary figures and images for: ﻿Molecular phylogeny of Nipponacmea (Patellogastropoda, Lottiidae) from Japan: a re-evaluation of species taxonomy and morphological diagnosis
Source: Zookeys. 2022 Feb 25;1087:163–98. doi: 10.3897/zookeys.1087.78193 (PMC8897378; doi:10.3897/zookeys.1087.78193)

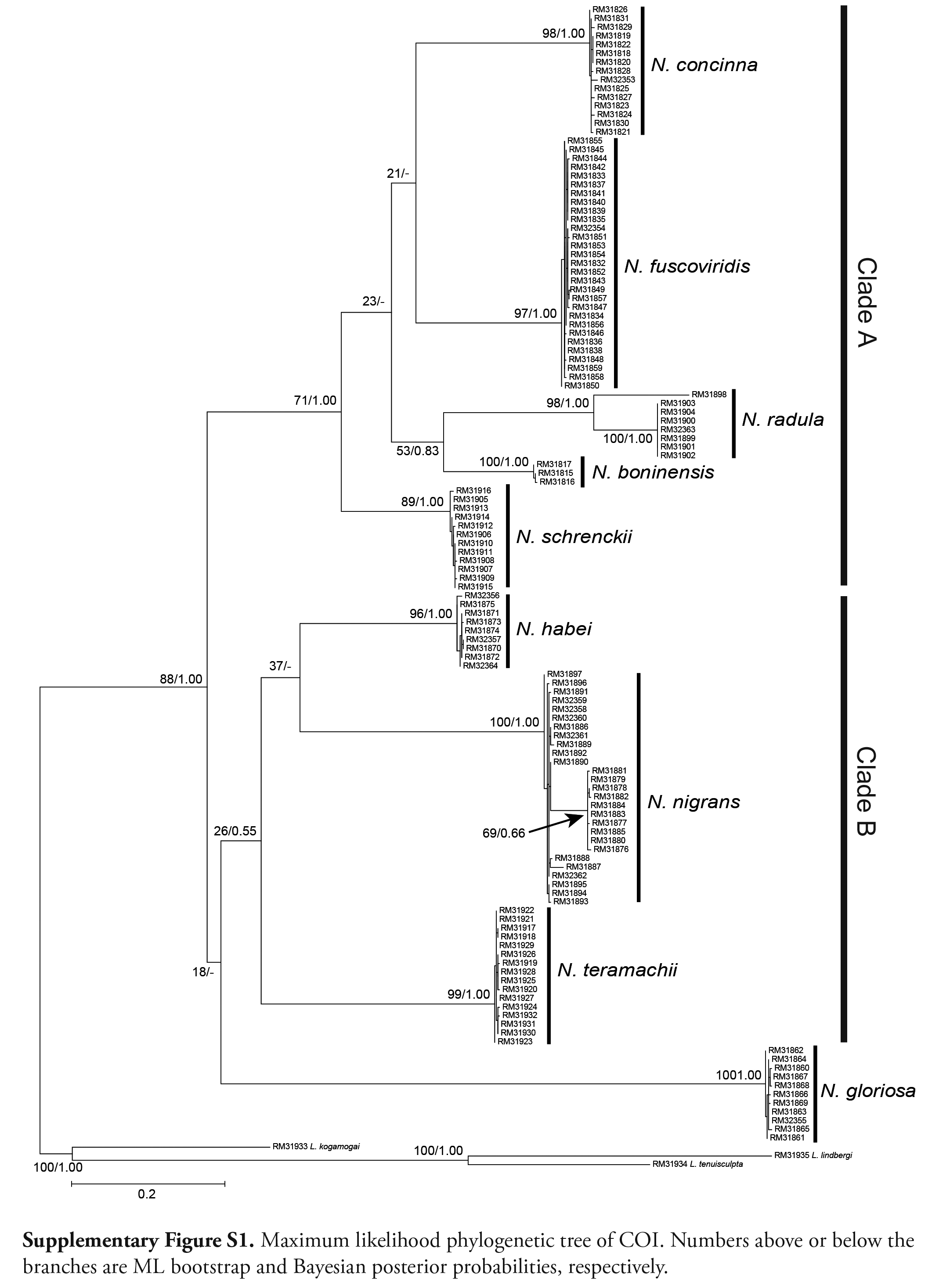

Supplement: Supplementary material 1 — Figure S1 [file zookeys-1087-163-s001.jpg]

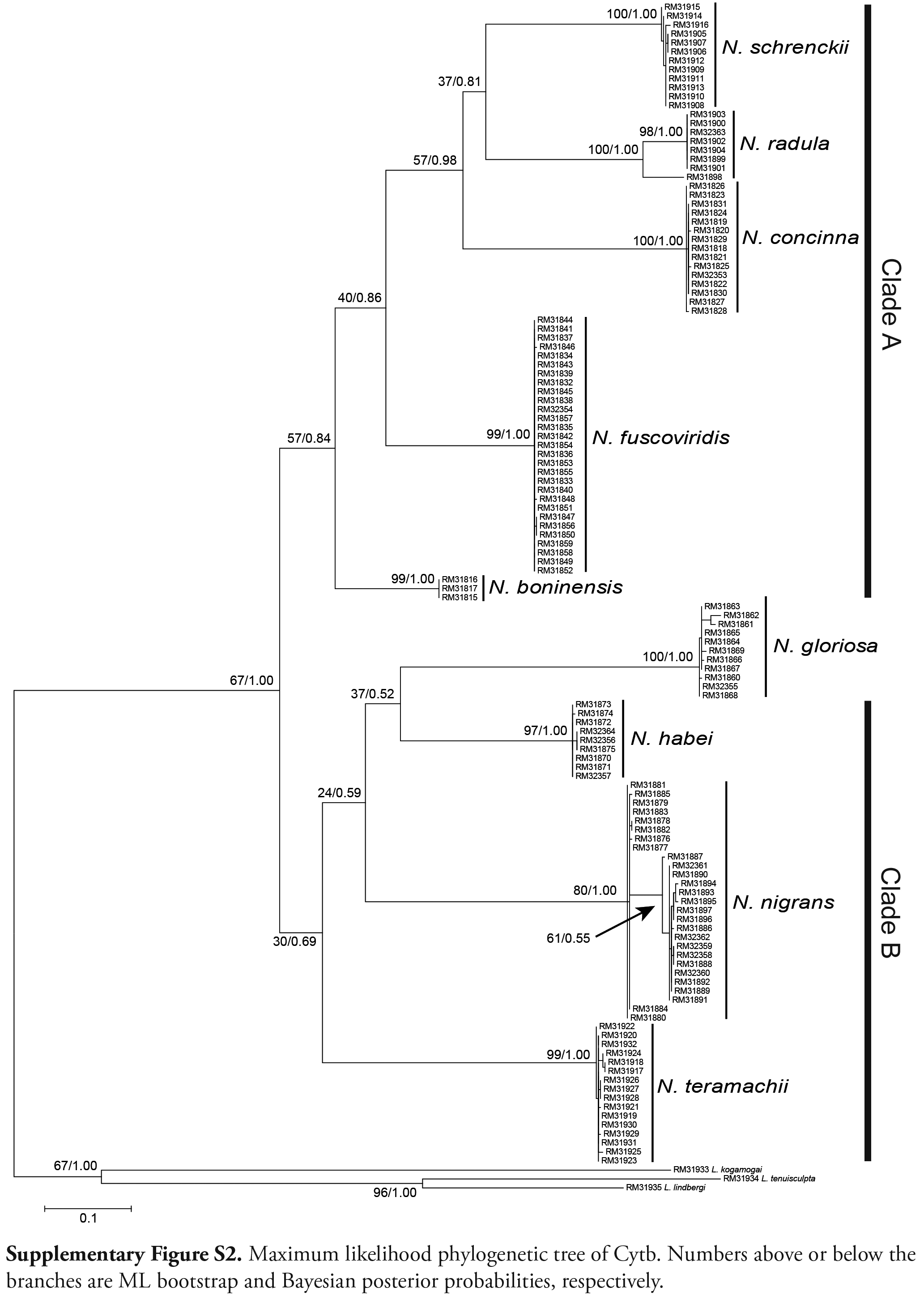

Supplement: Supplementary material 2 — Figure S2 [file zookeys-1087-163-s002.jpg]

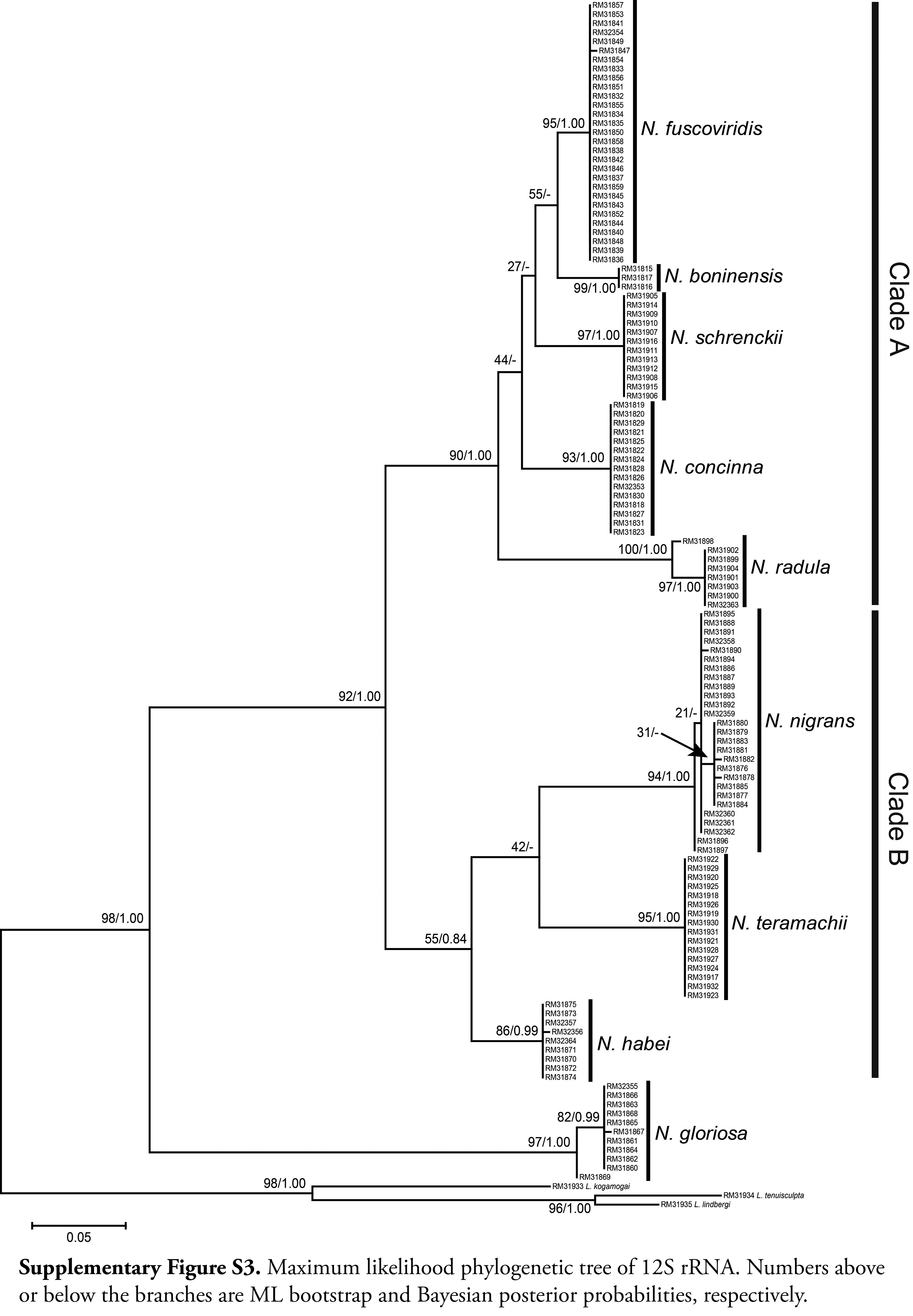

Supplement: Supplementary material 3 — Figure S3 [file zookeys-1087-163-s003.jpg]

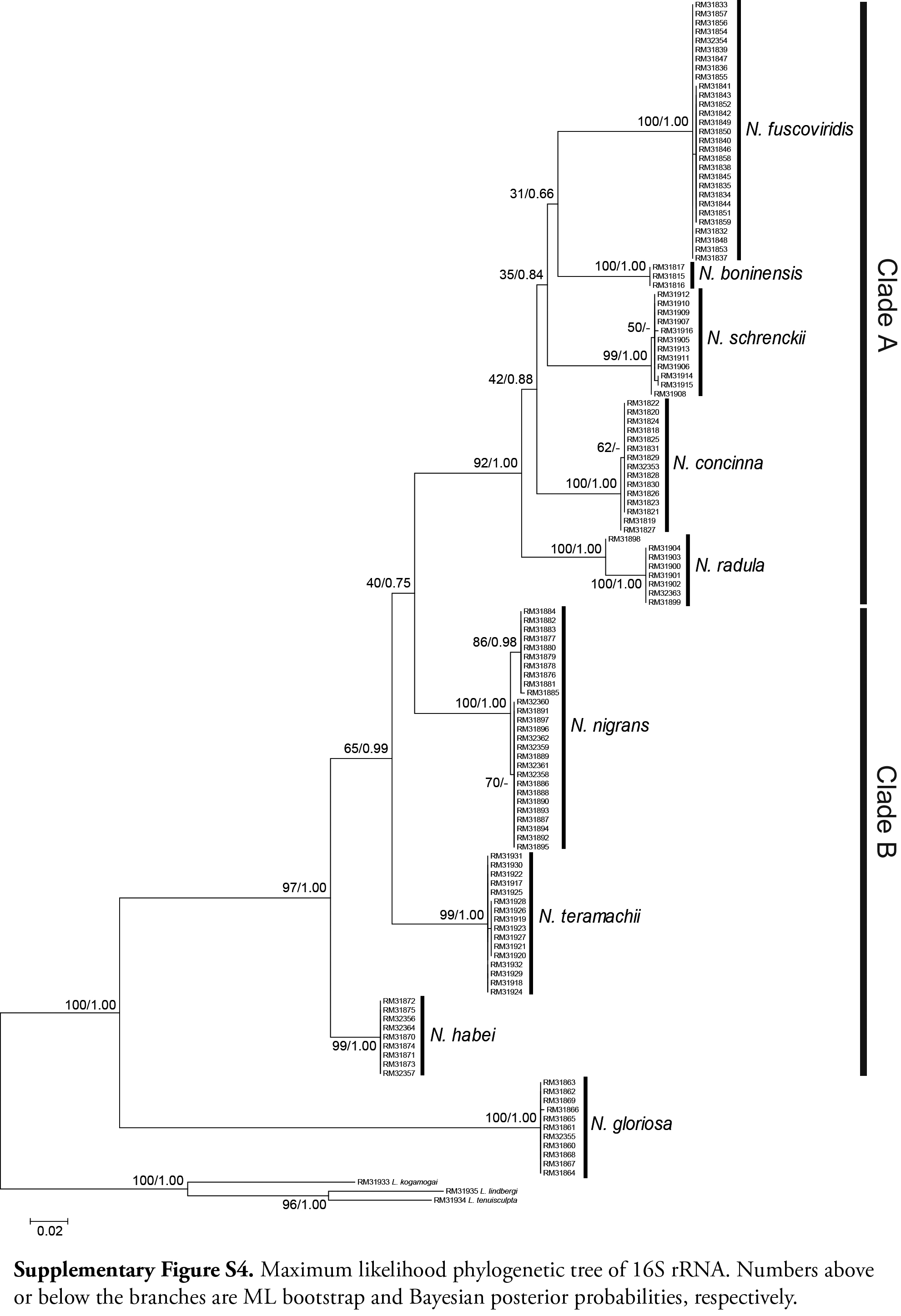

Supplement: Supplementary material 4 — Figure S4 [file zookeys-1087-163-s004.jpg]
